# Supplementary material for: Pathways of emergency care for severely ill children in Nigerian and Ugandan hospitals: A process mapping study
Source: PLoS Med. 2026 May 6;23(5):e1004832. doi: 10.1371/journal.pmed.1004832 (PMC13166908; doi:10.1371/journal.pmed.1004832)
Supplement: S1 Appendix — Table A. Deductive codes, following steps of the patient journey through a health facility. Table B. Description of facilities that align with Archetype A. Table C. Description of facilities that align with Archetype B. Table D. Description of facilities that align with Archetype C. Table E. Description of facilities that align with Archetype D. Table F. Initial assessment, treatment and admission locations for neonates (<29 days). Table G. Management of ‘special’ populations: older children, trauma and surgical conditions. Table H. Quotes from focus group discussions with researchers, organised under categories that explain steps in the patient journey through a facility. Table I. Characteristics of researchers participating in focus group discussions in Uganda and Nigeria. Figure A. Example of the evolution of process maps over time, from simple spatial maps of facility organisation (A) to conceptual maps of processes (B). Maps were supplemented by narrative descriptions. Text A. Researcher positionality. Text B. Process mapping data collection tool. Text C. Focus group discussion guide. Text D. Consent procedures and resources. (DOCX) [file pmed.1004832.s001.docx]

# Supporting information – Appendix S1

Mapping processes for the emergency care of severely unwell children in secondary and tertiary facilities in Nigeria and Uganda.

Contents

[Supporting information – Appendix S1 1](#_Toc228444217)

[Table A. Deductive codes, following the steps of the patient journey 2](#_Toc228444218)

[Table B. Description of facilities that align with Archetype A 3](#_Toc228444219)

[Table C. Description of facilities that align with Archetype B 4](#_Toc228444220)

[Table D. Description of facilities that align with Archetype C 7](#_Toc228444221)

[Table E. Description of facilities that align with Archetype D 10](#_Toc228444222)

[Table F. Initial assessment, treatment and admission locations for neonates (<29 days) 11](#_Toc228444223)

[Table G. Management of ‘special’ populations 16](#_Toc228444224)

[Table H. Quotes from focus group discussions with researchers 18](#_Toc228444225)

[Table I. Characteristics of researchers participating in focus group discussions 25](#_Toc228444226)

[Figure A. Example of the evolution of process maps over time. 27](#_Toc228444227)

[Text A. Researcher positionality 28](#_Toc228444228)

[Text B. Process mapping data collection tool 29](#_Toc228444229)

[Text C. Focus group discussion guide 39](#_Toc228444230)

[Text D. Consent – procedures and resources 41](#_Toc228444231)

[Staff Information and Consent Form 43](#_Toc228444232)

[Consent Form 46](#_Toc228444233)

## Table A. Deductive codes, following the steps of the patient journey through a health facility

| **Deductive code** | **Description** |
| --- | --- |
| Facility Navigation | How patients and caregivers move through the facility, including entry points, registration, and department transitions. |
| Facility Infrastructure | Physical and structural elements of the healthcare facility, including ward divisions, equipment availability, and layout. |
| Initial Assessment and Treatment (Triage) | Processes for identifying patient conditions, prioritising care, and initial clinical actions. |
| Administration and Payment | Tasks related to patient registration, records management, and financial transactions. |
| Oxygen Infrastructure | Availability, functionality, and use of oxygen delivery systems, including cylinders, concentrators, and pulse oximetry. |
| Monitoring | Processes for patient monitoring, including documentation of vitals and treatment progress. |
| Referral Processes | Steps and logistics involved in referring patients to higher-level facilities. |
| Role of Caregivers | Involvement of caregivers in transport, decision-making, and financial responsibilities. |
| Staffing – Challenges, Changes, Adaptations | Issues and adaptations related to staffing shortages, task-shifting, and workforce dynamics. |
| Role Delineation (of HCW) | How roles and responsibilities are defined and implemented for healthcare workers. |
| Communication | Flow of information between healthcare workers, patients, and caregivers. |
| Change over time | Changes noted between first and second round of data collection |

## Table B. Description of facilities that align with Archetype A

| **Facility** | **Entry and initial assessment** | **Initial treatment** | **Admission** |
| --- | --- | --- | --- |
| **HF3**  Uganda | **In-hours and after-hours**  **Where:**  OPD (0800-1700).  **Who and how:**  Triage conducted by non-clinical triage assistants, with basic training in triage and vital sign assessments, who also serve as interpreters for a large refugee population. | Recognised emergency cases are moved to a procedure room for stabilisation, with emergency equipment and supplies for adults and children. The procedure room, dispensary and registration desk are collocated. Management plans by clinical officers, enacted by nurses. | Paediatric IPD. |
|  | **After-hours**  Children are directed to paediatric IPD, where registration, assessment and stabilisation is conducted by nurses in a ‘duty room’. | Paediatric IPD. Nurse run over-night. Clinical officer and doctor on-call. |  |
| **HF5**  Uganda | **In-hours and after-hours**  OPD (24 hour). Triage conducted by nurses. separate medical (2 beds) and surgical (1 bed) emergency rooms in OPD. | Some basic emergency medications and oxygen are available in the emergency rooms, prescribed by clinical officers and administered by outpatient nurses. Doctor stationed in admission ward, called for emergencies. | Paediatric IPD. Shared staffing and equipment with adult wards. |
| **HF9**  Uganda | **In-hours**  **Where:**  OPD (0800-1700).  **Who and how:**  Triage in HF9 is conducted by nurses, in order of patient arrival. There is some existing organisation for triage (e.g. colour coded physical spaces). Decisions on severity are made by nurses who prioritise referred patients, and caregiver concern. | Emergency room in OPD for recognised critically unwell patients, equipped with oxygen and pulse oximeters. Management plans by clinical officers, enacted by nurses. | Paediatric IPD. |
|  | **After-hours**  Children are directed to paediatric IPD | Paediatric IPD. |  |

HF: health facility; OPD: outpatient department; IPD: inpatient department

## Table C. Description of facilities that align with Archetype B

| **Facility** | **Initial assessment** | **Initial treatment** | **Admission** |
| --- | --- | --- | --- |
| **HF1**  Uganda | **In-hours**  **Where:** 29 days–5 y enter via OPD (0800-1800);  ≥6 y enter via ED.  **Who and how:**  OPD staffed by nurses who register, check vital signs and send for clinical officer review.  ED: ETAT colour-coded triage system with red/yellow/green spaces. Triage by nurses, and then reviewed by doctor. | OPD does not have emergency therapies. Transfer directly to paediatric IPD (29 days–5 y) or ED (≥6 y) for treatment.  Prescribed by clinical officers in OPD or nurse initiated in IPD. Doctor available for rounds and on-call.  In ED, medical review and plan. Administration by nurses. | 29 days–5y paediatric IPD;  ≥6y. ED or adult ward.  Patients can remain in ED for up to 24 hours prior to discharge or transfer to inpatient ward. |
|  | **After-hours**  29 days–5 y go directly to paediatric IPD  ≥6 y enter via ED |  |  |
| **HF2**  Uganda | **In-hours**  **Where:** 0–5 y enter via OPD (0800-1800);  ≥6 y enter via ED.  **Who and how:**  OPD staffed by nurses or community extension officers who conduct triage, check vital signs. Sent to clinical officer for further assessment. If concerns about severe illness requiring urgent treatment at triage, children are sent directly to wards for resuscitation by IPD nurses. | OPD does not have emergency therapies. Transfer directly to paediatric IPD (0–5 y), general male/female wards (≥6 y) or theatre for treatment. | 0-5y paediatric IPD.  ≥6 y general male/female wards. |
|  | **After-hours**  0–5 y go directly to paediatric IPD.  ≥6 y to general male/female wards. |  |  |
| **HF4**  Uganda | **In-hours**  **Where:** OPD (0800–1700)  **Who and how:**  Nurses check vital signs. Assessment and plan documented by clinical officers. If concerns about severe illness requiring urgent treatment at triage, children are sent directly to wards for resuscitation by ward nurses. | OPD does not have emergency therapies. Transfer directly to paediatric IPD (29 days–12 y) or male/female wards (≥13 y) for treatment.  Treatments prescribed by clinical officers in OPD, nurse initiated in IPD, or prescribed following medical review in IPD. | 29d-12y, paediatric IPD.  ≥13y, male/female ward.  Paediatric IPD does not have oxygen, so if oxygen therapy needed, admission to male/female ward. |
|  | **After-hours**  29d-12y, paediatric IPD.  ≥13y, male/female ward.  Nurse assessment and plan. Doctor on-call. |  |  |
| **HF6**  Uganda | **In-hours**  **Where:** OPD (0800–1700).  **Who and how:**  Nurse at triage does registration and triage, followed by clinical officer review. OPD has stabilisation room for assessment of very unwell patients. Further assessment and plan by clinical officer. No formal triage tool. | OPD has a stabilisation room, but emergency treatments only available and provided in IPD or theatre. Administration by nurses. | 29d–11y paediatric IPD.  ≥12y adult male/female wards.  Paediatric and adult wards are separate sections of the same general ward. |
|  | **After-hours**  29 days–11 y go directly to paediatric IPD.  ≥12y present to adult male or female wards.  Initial assessment and treatment by ward nurses. |  |  |
| **HF7**  Uganda | **In-hours**  **Where:** OPD (0800–1800), dedicated assessment space.  **Who and how:**  Nurses and receptionists record biodata and check vital signs. Assessment and plan documented by clinical officers or doctors (one covering OPD and IPD). If concerns about severe illness requiring urgent treatment at triage, children are sent directly to wards for resuscitation by ward nurses. | OPD does not have emergency therapies. Transfer directly to inpatient wards for treatment.  Prescription by doctor/clinical officer and administration by ward nurses. | 29 days–10y go directly to paediatric IPD.  ≥11y present to adult male or female wards. |
|  | **After-hours**  29 days–10y go directly to paediatric IPD.  ≥11y present to adult male or female wards.  Initial assessment and treatment by ward nurses, then review by clinical officer/doctor |  |  |
| **HF8**  Uganda | **In-hours**  **Where:** OPD (0800–2000).  **Who and how:**  Nurses and receptionists record biodata and check vital signs. Assessment and plan documented by clinical officers. If concerns about severe illness requiring urgent treatment at triage, children are sent directly to wards for resuscitation by ward nurses. | OPD does not have emergency therapies. Transfer directly to inpatient wards for treatment.  Prescription by clinical officer (or nurse initiation) and administration by ward nurses. | 2m–12y paediatric IPD.  ≥13y adult male/female wards. |
|  | **After-hours**  OPD if on-call clinical officer on-site. Otherwise, direct to admission wards: 2m–12y paediatric IPD.  ≥13y adult male/female wards. |  |  |
|  | **After-hours**  Paediatric OPD closed. Patients directed to paediatric IPD. |  |  |
| **HF13**  Nigeria | **In-hours:**  **Where:** OPD (08:00–16:00); also separate ED.  **Who and how:** OPD: nurse takes vitals, medical officers assess and develop treatment plan. If concerns about severity, sent to ED.  In ED, one nurse on shift receives patients and calls the on-call doctor (covers entire facility). | OPD does not have emergency therapies. Emergency treatments in ED. Oxygen borrowed from maternity (emergency unit has an empty cylinder/no concentrator). Medications prescribed by medical officer and administered by the nurse. | ED can hold patients for up to ~24 hours.  Combined IPD admits all ages. Includes 2 baby cots. No oxygen available on ward. |
|  | **After-hours:**  Present to ED. Emergency nurse conducts informal triage and alerts the on-call doctor. |  |  |
| **HF18**  Nigeria | **In-hours:**  **Who and how:**  OPD nurse initially assesses, followed by medical review. One is a paediatrician available three days a week. | OPD does not have emergency therapies. Transfer to inpatient ward for treatment. | Combined IPD for all ages. |
|  | **After-hours:**  Present to combined IPD ward. |  |  |

HF: health facility; OPD: outpatient department; IPD: inpatient department ; ED: emergency department; ICU: intensive care unit.

## Table D. Description of facilities that align with Archetype C

| **Facility** | **Initial assessment** | **Initial treatment** | **Admission** |
| --- | --- | --- | --- |
| **HF11**  Uganda | **In-hours**  **Where:**  Children enter OPD triage (called ‘OPD triage’) and are sent to ED for emergency assessment and treatment.  **Who and how:**  Triage by a nurse. Further assessment in ED by an intern doctor or clinical officer. | In ED. Interns/clinical officers prescribe; nurses cannulate and give medications. | 29 d–12 y: Paediatric IPD (HDU area for the critically ill).  >12 y: Adult medical/surgical wards (HDU areas available). |
|  | **After-hours**  Triage area closes ~1400, after which, streaming directly to ED. |  |  |
| **HF14**  Nigeria | **In-hours and after-hours**  **Where:** ED is the entry point for paediatric emergencies/referrals. Paediatric OPD runs concurrently during in-hours, and refers unwell children to ED.  **Who and how:**  Initial assessment by nurse. Further assessment by general doctor. | In ED. Nurse and general doctor. | All children requiring admission beyond a few hours referred to another facility, as paediatric IPD is non-functional due to renovation. |
| **HF15**  Nigeria | **In-hours**  **Where:**  Children enter OPD triage (called ‘OPD triage’) and are sent to ED for emergency assessment and treatment.  **Who and how:**  Nurses or CHEW by general appearance and clinical signs. | Paediatric ED. Interns/clinical officers prescribe; nurses cannulate and give medications. | Paediatric ED. |
|  | **After-hours**  Streaming directly to ED. |  |  |
| **HF16**  Nigeria | **In-hours and after-hours**  Paediatric ED runs 24-hours.  **Who and how:**  Triage nurse and medical assessment. | In ED. Nurses, junior and specialist doctors. | Paediatric IPD. |
| **HF17**  Nigeria | **In-hours and after-hours**  Paediatric ED runs 24-hours.  **Who and how:**  Triage nurse, CHEW, and medical assessment. | **Where and who:**  In ED. Nurse and general doctor. | Paediatric IPD. |
| **HF19**  Nigeria | **In-hours and after-hours**  **Where:** Paediatric ED is the entry point for paediatric emergencies/referrals. Paediatric OPD runs concurrently during in-hours, and refers unwell children to ED.  **Who and how:**  Initial assessment by nurse. Further assessment by general doctors. | In paediatric ED. Nurse and general doctor. | Short-stay admission to paediatric ED. Longer admissions to paediatric IPD. |
| **HF20**  Nigeria | **In-hours and after-hours**  **Where:** Paediatric ED is the entry point for paediatric emergencies/referrals. Paediatric OPD runs concurrently during in-hours, and refers unwell children to ED.  **Who and how:**  Initial assessment by nurse/nurse assistant. Further assessment by general doctors. | In paediatric ED. Nurse and general doctor. | Paediatric IPD. |
| **HF21**  Nigeria | **In-hours and after-hours**  **Where:** Paediatric ED is the entry point for paediatric emergencies/referrals. Paediatric OPD runs concurrently during in-hours, and refers unwell children to ED.  **Who and how:**  Initial assessment by nurse/nurse assistant. Further assessment by general doctors. | In paediatric ED. Nurse and general doctor. | Paediatric IPD. |
| **HF22**  Nigeria | **In-hours and after-hours**  **Where:** Paediatric ED is the entry point for paediatric emergencies/referrals. Paediatric OPD runs concurrently during in-hours, and refers unwell children to ED.  **Who and how:**  Initial assessment by nurse/CHEW. Further assessment by general doctors. | In paediatric ED. Nurse and general doctor. | Paediatric ED.  *Paediatric IPD under construction. |
| **HF23**  Nigeria | **In-hours and after-hours**  **Where:** Paediatric ED is the entry point for paediatric emergencies/referrals. Paediatric OPD runs concurrently during in-hours, and refers unwell children to ED.  **Who and how:**  Initial assessment by nurse/nurse assistant. Further assessment by doctors. | In paediatric ED. Nurse and general doctor. | Paediatric IPD. |
| **HF24**  Nigeria | **In-hours and after-hours**  **Where:** ED is the entry point for paediatric emergencies/referrals. Paediatric OPD runs concurrently during in-hours, and refers unwell children to ED.  **Who and how:**  Initial assessment by nurse/nurse assistant. Further assessment by doctors. | In ED. Nurse and general doctor. | Paediatric IPD. |
| **HF25**  Nigeria | **In-hours and after-hours**  **Where:** ED is the entry point for paediatric emergencies/referrals. Paediatric OPD runs concurrently during in-hours, and refers unwell children to ED.  **Who and how:**  Initial assessment by nurse/nurse assistant. Further assessment by doctors. | In ED. Nurse and general doctor. | Paediatric IPD. |
| **HF26**  Nigeria | **Where:** ED is the entry point for paediatric emergencies/referrals. Paediatric OPD runs concurrently during in-hours, and refers unwell children to ED.  **Who and how:**  Initial assessment by nurse/nurse assistant. Further assessment by doctors. | In ED. Nurse and general doctor. | Paediatric IPD. |

CHEW: community health extension officer; HF: health facility; ED: emergency department; ICU: intensive care unit; OPD: outpatient department; IPD: inpatient department.

## Table E. Description of facilities that align with Archetype D

| **Facility** | **Initial assessment** | **Initial treatment** | **Admission** |
| --- | --- | --- | --- |
| **HF10**  Uganda | **In-hours**  **Where:**  Patients can present directly to a triage desk located outside the paediatric IPD (intended Archetype), or to the adult ED.  **Who:**  Triage by nurse, clinical officer or intern doctor.  In ED, assessment by adult ED nurse, management by general ED doctor. | Acute care unit (dedicated space within paediatric IPD). | Paediatric IPD. |
| **HF12**  Uganda | **In-hours**  **Where:**  Patients can present directly to paediatric IPD (intended Archetype), paediatric OPD or ED.  **Who:**  In paediatric IPD initially assessed by doctor (medical officer) in a dedicated room. Triage by clinical signs and risk factors (e.g. neonates given priority). Checks vital signs, and prescribes emergency treatment. Administration by nurse and admission to ward. High acuity space within the ward available for severely unwell children.  In OPD, initial triage by nurse (by observation, no formal system), then assessed by clinical officer who staffs the paediatric and general OPD. Children requiring admission sent to paediatric IPD.  In ED, patients assessed and triaged by nurses and doctors (no formal triage). Care for patients with trauma, and older children (≥15y). | OPD does not have emergency therapies. Transfer directly to paediatric IPD.  Prescribed by doctor, administered by nurse in admission room or following admission to ward bed.  Children (<15y) presented to ED sent to paediatric IPD. If older, or presenting with trauma, therapies prescribed by ED doctor, administered by nursing staff in ED bay. | 29 days–14y  paediatric IPD. HDU section if oxygen needed. Step down to critical unit, then general ward.  <15y adult wards.  Separate paediatric surgical ward, and paediatric oncology department.  Mixed adult/paediatric ICU that can provide mechanical ventilation for neonates and children. |
|  | **After-hours**  29 days–5 y go directly to paediatric IPD  ≥6 y enter via ED |  |  |

HF: health facility; ED: emergency department; ICU: intensive care unit; OPD: outpatient department; IPD: inpatient department.

## Table F. Initial assessment, treatment and admission locations for neonates (<29 days)

*Neonatal (<29 days) pathways were on the whole different to those of older children, with distinct pathways for inborn and outborn newborns. Broadly, variations of these were:*

*-Dedicated NNUs, 15 facilities (shaded orange in Table F)*

*-Care led by maternity units, provided by midwives in labour or post-natal wards. Found in small Ugandan facilities (shaded green in Table F).*

*- Pathways similar to that of children, with stabilisation in ED and admission to paediatric or general ward (shaded purple in Table F).*

*-No neonatal services, with referral to another facility. Three facilities in Nigeria (shaded grey in Table F).*

| **Facility** | **Entry and initial assessment** | **Initial treatment** | **Admission** |
| --- | --- | --- | --- |
| **HF1**  **Uganda** | **In-hours and after-hours**  Inborns stabilised in resuscitation bed of labour suite or OT, then transferred to NNU.  Outborns present directly to NNU.  Inborn initially resuscitated by midwives in labour suite. NNU is predominantly nurse led, with less regular medical reviews. Assessment includes vital signs (and SpO2). | Inborns: Labour suite resuscitation space and NNU  Outborns: NNU | NNU. |
| **HF3**  **Uganda** | Inborns stabilised in OT or labour suite, and then transferred for ongoing management in NNU.  Outborns assessed in OPD. | Inborns: OT/labour suite, then NNU  Outborns: OPD procedure room then NNU. | NNU. |
|  | **After-hours**  In-hours Archetype for inborns.  Outborns present directly to NNU. |  |  |
| **HF5**  **Uganda** | **In-hours and after-hours**  **Where:**  Inborns moved to NNU.  Outborns:  OPD (24 hour). Triage conducted by nurses. | Inborns: NNU  Outborns: Emergency room in OPD, then moved to NNU. | NNU. |
| **HF8**  **Uganda** | **In-hours and after-hours**  Inborn from labour suite or OT sent to NNU.  Outborn registered in OPD and sent to NNU.  **After-hours**  In-hours Archetype for inborns.  Outborns present directly to NNU. | NNU. | NNU. |
| **HF9**  **Uganda** | **In-hours and after-hours**  Inborn sent to NNU (main).  Outborn sent to NNU (annex). | NNU (main or annex sections). | NNU (main or annex sections). |
| **HF10**  **Uganda** | **In-hours and after-hours**  Inborn and outborn moved to NNU. | NNU. | NNU. |
| **HF11**  **Uganda** | **In-hours:**  Inborn sent directly to NNU (main)  Outborn initially assessed in OPD, then sent to NNU (annex) | Inborn NNU (main).  Outborn NNU (annex). | Inborn NNU (main).  Outborn NNU (annex). |
|  | **After-hours:**  In-hours Archetype for inborns.  Outborn initially assessed in ED, then sent to NNU (annex) |  |  |
| **HF12**  **Uganda** | **In-hours and after-hours**  Inborn from labour suite/OT to paediatric admission room (space within paediatric IPD).  Outborn sent from OPD/ED to paediatric admission room. | Paediatric admission room | NNU |
| **HF20**  **Nigeria** | **In-hours and after-hours**  Inborns from OT or labour suite stabilised in a dedicated space in labour suite.  Outborn: Paediatric ED. | Inborn: NNU (inborn).  Outborn: paediatric ED (if short stay). | Inborn: NNU (inborn).  Outborn: NNU (outborn). |
| **HF21**  **Nigeria** | **In-hours and after-hours**  Inborns from OT or labour suite stabilised in a dedicated space in labour suite.  Outborn: Paediatric ED. | Inborn: NNU (inborn).  Outborn: paediatric ED (if short stay). | Inborn: NNU (inborn).  Outborn: NNU (outborn). |
| **HF22**  **Nigeria** | Inborn and outborn sent to NNU. | NNU. | NNU. |
| **HF23**  **Nigeria** | **In-hours and after-hours**  Inborns from OT or labour suite stabilised in a dedicated space in labour suite.  Outborn: Paediatric ED. | Inborn: NNU (inborn).  Outborn: paediatric ED (if short stay). | Inborn: NNU (inborn).  Outborn: NNU (outborn). |
| **HF16**  **Nigeria** | **In-hours and after-hours**  Inborns from OT or labour suite stabilised in a dedicated space in labour suite.  Outborn: Paediatric ED. | Inborn: NNU (inborn).  Outborn: paediatric ED. | Inborn: NNU (inborn).  Outborn: NNU (outborn). |
| **HF25**  **Nigeria** | Inborns from OT or labour suite stabilised, before transfer to NNU (inborn).  Outborn: present directly to NNU (outborn). | NNU (inborn and outborn). | NNU (inborn and outborn). |
| **HF26**  **Nigeria** | Inborns from OT or labour suite stabilised, before transfer to NNU (inborn).  Outborn: present directly to NNU (outborn). | NNU (inborn and outborn). | NNU (inborn and outborn). |
| **HF2**  **Uganda** | **In-hours and after-hours**  Inborns stabilised in OT or labour suite, and then transferred for ongoing management in post-natal ward.  Outborns assessed in OPD then sent to paediatric IPD for management.  Nurse-led, medical reviews. Depending on availability, also clinical officer or medical review. | Inborns: OT/labour suite.  Outborns: Paediatric IPD. | Paediatric IPD. |
|  | **After-hours**  In-hours Archetype for inborns.  Outborns present directly to paediatric IPD. |  |  |
| **HF4**  **Uganda** | **In-hours and after-hours**  Inborns stabilised in resuscitation bed of labour suite or OT. If weaned off oxygen, ongoing care in post-natal ward. NNU.  Outborns assessed in OPD, then sent to labour suite (if needs oxygen) or paediatric IPD for ongoing care.  Inborns: midwife led care, with medical reviews.  Outborns: initially assessed by clinical officers (in-hours) then midwife or nurse led care depending on disposition. | Inborns: Labour suite or OT.  Outborns: Labour suite. | Inborns: Post-natal ward (if off O2), transfer if needing oxygen beyond 6 hours.  Outborns: Labour suite if needs oxygen, and transfer if ongoing oxygen need beyond 6 hours. Paediatric IPD if off oxygen, |
|  | **After-hours**  In-hours Archetype for inborns.  Outborns present directly to maternity. |  |  |
| **HF6**  **Uganda** | **In-hours and after-hours**  **Where:**  Inborns from OT or labour suite stabilised in a dedicated space in labour suite.  Outborns: register in OPD and directed to maternity.  Midwife led care, with medical reviews. | Labour suite | Post-natal ward |
|  | **After-hours**  In-hours Archetype for inborns.  Outborns present directly to maternity. |  |  |
| **HF7**  **Uganda** | **In-hours and after-hours**  **Inborn and outborn**  Resuscitation space in labour suite or NNU. But NNU usually not used as further away from maternity.  Shared staffing with maternity. Midwife led care, with medical reviews. | Post-natal ward. | Post-natal ward. |
| **HF14**  **Nigeria** | Present to ED, referred to another facility. | Referred to another facility. | Referred to another facility. |
| **HF15**  **Nigeria** | Present to OPD (in-hours) or ED (after-hours), referred to another facility. | Referred to another facility. | Referred to another facility. |
| **HF17**  **Nigeria** | Present ED, referred to another facility. | Referred to another facility. | Referred to another facility. |
| **HF13**  **Nigeria** | **In-hours:**  OPD (08:00–16:00); also separate ED.  OPD: nurse takes vitals, medical officers assess and develop treatment plan. If concerns about severity, sent to ED.  In ED, one nurse on shift receives patients and calls the on-call doctor (covers entire facility). | Inborn: maternity or OT  Outborn: Emergency or Paediatric IPD | Inborn: Maternity.  Outborn: General (combined) ward. |
|  | **After-hours:**  Present to ED. Emergency nurse conducts informal triage and alerts the on-call doctor. |  |  |
| **HF18**  **Nigeria** | **In-hours**  OPD | OPD does not have emergency therapies. Transfer to inpatient ward for treatment. | Combined IPD. |
|  | **After-hours**  Present to combined IPD ward. |  |  |
| **HF19**  **Nigeria** | **In-hours and after-hours**  Inborn stabilised in OT/labour ward and then sent to either NNU or Paediatric ED.  Outborn to Paediatric ED. | Inborn: NNU or paediatric ED.  Outborn: Paediatric ED (if short stay). | NNU or paediatric ED. |
| **HF24**  **Nigeria** | Inborns from OT or labour suite stabilised, before transfer to NNU.  Outborn: ED is the entry point for paediatric emergencies/referrals. | Inborn: NNU.  Outborn: ED | Inborn: NNU.  Outborn: Paediatric IPD. |

Dedicated NNU (orange shading), maternity led (green shading), no neonatal services (grey shading) and shared pathways with children (purple shading). ED: emergency department; IPD: inpatient department; NNU: neonatal unit; OT: operating theatre.

## Table G. Management of ‘special’ populations: older children, trauma and surgical conditions

| **Patient population** | **Pathway** |
| --- | --- |
| Older children or trauma | *Share adult services*  “Sick children coming in to the general outpatient department are assessed by clinical officers; patients clinically determined to be needing admission are transferred to the Accidents and Emergency unit (for children 13-15 years and < 12 years with surgical/trauma conditions)...” HF10, Uganda  “Patients with trauma and above 12-15 years old go through adult accident and emergency. After being seen by a doctor, if there is a need for surgery, they are transferred to either the ward first or directly to the theatre, depending on the condition and severity. After surgery, patients are moved to the adult male/ female ward for recovery before discharge home.” HF20, Nigeria |
| Surgical conditions | *Share adult services*  “Children with surgical conditions coming in as referral from peripheral health facilities or those identified at the paediatric outpatient department by the clinical officer are moved to the emergency department where they are triaged following Emergency Triage Assessment and Treatment (ETAT) guidelines, assessed by the nurses and reviewed by the medical doctor. The medical doctor carries out physical assessment, triages patients who are to be operated at the main operating theatre and refers patients to higher level facilities. Emergency surgical patients are moved directly to the theatre by nurses.” HF1, Uganda  “Children with surgical conditions come in through the hospital gate and move to the triage area where they are registered by the nurses (biodata, pulse oximetry, and anthropometry assessments). The patients thereafter are moved to the medical clinical officer's consultation rooms where they are assessed and diagnosed. Patients with non-life-threatening surgical conditions are then admitted to their respective wards where they are reviewed by the medical doctor on duty and the anaesthetic officer and have their pre-operative medication administered by the nurses on duty. Patients are then moved to either the operating theatre for surgical intervention or referred to higher-level facilities. Patients with emergency surgical conditions are moved to the operating theatre on arrival to the health facility or first resuscitated from their respective wards and then referred to higher-level health facilities.” HF2, Uganda  “Surgical patients: For paediatric surgical cases, patients are seen at the surgical clinic and admitted to the surgical ward prior to and after surgery. If the patient was brought in as an emergency, the patient is taken directly to the theatre and, after surgery, to the surgical ward.” HF23, Nigeria  “Child is taken off the vehicle, moved by stretcher through triage Area to Emergency/casualty section. At Emergency, the child is taken to the tent. It’s in the tent where surgical emergency cases are handled. The child is received by a Nurse and Assessed by an Intern Doctor. There's an Intern Doctor who is responsible for reviewing patients with Surgical conditions. The child receives first management from the tent and after is stabilized, the child is sent to ward; however, if child needs surgery, child is still sent to ward and it is from the ward where the child is taken to theatre.” HF11, Uganda |

## Table H. Quotes from focus group discussions with researchers, organised under categories that explain steps in the patient journey through a facility

| **Category** | **Sub-category and illustrative quotes** |
| --- | --- |
| Perceived acuity can expedite patient journey | *Through general appearance*  “They [healthcare workers] would **look in the patients who have come or they would even identify them when they are still in the local site or entering and they realise that child is very sick**. For the case of [HF9] they would either send that child directly to the paediatric ward or to a clinical room to be reviewed by a clinical officer.” FGD, HF9, Uganda.  *Through referral letter*  “Then for facilities like [HF11], usually when the kid is sick, it is **identified from the main gate by the security guards**. And most of the time, the referrals, they come when they are really sick. **And when they come with the referral letters, they just go straight to the emergency department**. FGD, HF11, Nigeria. |
| Facility navigation complicated by change | “There are **no staff at triage during the night**. The emergency cases coming to the facility at night are not triaged or assessed on arrival. Instead, they move directly to the inpatient ward for first assessment and care by the inpatient staff.” HF2, Uganda.  “If the sick child comes beyond 2pm, at that time triaging area is closed, the very sick child moves straight to emergency room since triaging area closes at around 2 pm. Patients who do not know where to go are directed by the gate man or [otherwise] ask anyone they find at OPD.” HF11, Nigeria. |
| Tacit knowledge | *Common knowledge*  “I can give an example, from [HF12]. So **the facility does not have a formal triage system**, but what they do is, when patients come through the gate, mostly in the community, **people know that for babies from 0 to around 10 or 11, they go directly to the paediatric ward.** They don't have to go to the emergency department of the hospital. So it's like **common knowledge**, and for those who do not know, go to the emergency, and then the triage staff for the emergency department sends them down to the paediatric ward.” FGD, HF12, Uganda.  “The neonates that are brought by ambulance, **the ambulance driver said he does not take the neonates to the triage area** or the ambulance room because he thinks, sorry, the emergency room, **because according to what he has seen, he says care is not good in that place**. So he takes them straight to the neonatal unit.” HF11, Uganda.  “He was asking about direction. Maybe indication on how to get to the unit you want to get to. Sometimes the lack of knowledge might affect that. Because they might tell you to go towards this direction. But **because you can't read what is written there. It is written in English. You won't be able to decipher** if that is the place you are supposed to go to.” FGD, HF18, Nigeria.  “Because some caregivers don't even know who is the nurse, who is the CHEW [community health extension worker], who is the doctor, or even the substaff [cleaners] they believe they are a doctor. So sometimes they used to even present their children to substaff there at the facility.” FGD, HF22, Nigeria |
| The role of caregivers – distress expedites care | “You see some caregivers coming, shouting, **"My child! My child!! Oxygen!!!"** There was one patient who followed. And from the gates, they were all shouting, "Oxygen! Oxygen!!"  So the nurses, like then, most of them left, **some were actually sitting, maybe the ones that were not busy. So they ran** to, like, help the child, drop the child on the resuscitation couch. So it depends on how the caregivers, how agitated they are.” FGD, HF26, Nigeria. |
| Barriers to implementation of triage systems | *Workload*  “But the healthcare workers will just do their work. What I observe is that all **their attention is to cover the patient that they have in their own because the facility has high inflow of patients.** So, even if there is an official system of triage there, I think they don't use to follow the system because of the high inflow of patients…”. FGD, HF26, Nigeria  *Caregiver perceptions*  “…I think it [triage systems] will not work because most of the caregivers who brought their children there, they believe that their children, the sickness of their children is not like, it's like, they believe that, for example, let's say I brought my child to the hospital. **So, I have the feeling like my child's sickness is far above that everyone there. So, I think my own has to be attended to.**” FGD, HF26, Nigeria.  *Unpredictable workforce*  “So as much as they had the triage system labelled clearly, they were not following it. But when I asked, I inquired why they did not follow, one staff said they were understaffed because you find that maybe you find that two staffs were attached to OPD and they depended heavily on volunteers who sometimes come and also sometimes they don't come.” FGD, HF7 |
| Approaches to triage | *By non-clinical staff*  “Some days, there's a nurse. Other days, there's not a nurse. **I noted there is an Ascari [security guard] who does the triage**.” FGD, HF2, Uganda  “As they are waiting, one sub-staff [cleaner] asked the caregivers about complain of their child so that to know which consultation table is she going [to] introduce them one after the other.” HF22, Nigeria  “The two receptionists do not have medical training background; however, they work at the triage desk; one on each duty shift (8 am to 6 pm) whereby they are responsible for capturing patient biodata (name, age, sex address, next of kin, telephone contact) and carrying out vitals assessment and recording the findings in patient files and outpatient register.” HF7, Uganda  “One substaff [cleaner] arrange the caregivers and their children and then introduce them one after the other to the nurses.” HF15, Nigeria  *Fever as concerning*  “What I've observed they [healthcare workers] do is they look at any patient presenting with **high fever**, that patient must be attended to first. **Though even if most of, they don't have the ETAT, like you said, displayed on the wall, they just know that on their own, even if they see [the poster], just like you say, most people might not even follow it.”** FGD, HF24, Nigeria.  *Absence of fever as reassuring*  “I was thinking like, because **she [the mother] was screaming like, my baby, my son need attention.** I am sure there is something really wrong with him. But since **the temperature was calm**, not that obvious like before, the nurses said, madam, when did you retrieve your folder? When did you retrieve your folder? The woman said, so I should wait here, till everybody. They said yes. And they do. **They follow, first come, first served.** When you retrieve your folder, that is when they attend to you.” FGD, HF18, Nigeria.  *First come, first served*  “Let me use [HF22] as our example. That facility has no official system of triaging at all. For example, they just do it, when it comes to healthcare workers in assessment, they just do it like first come, first serve, no matter the type of condition or something like that.” FGD, HF22, Nigeria. |
| Work outside scope of practice | *Community health workers taking on care of critical illness*  “Okay, you can find out, a community healthcare extension worker, that is **CHEWs, attended to serious critical issues**. Yes, critically ill-people. Like, it's supposed to be attended by a nurse or a doctor or a consultant.” FGD, HF22, Nigeria.  *Students taking on clinical roles*  “When students are present, they take up the roles of the nursing assistants. The nursing assistants are allocated around overseeing what they are doing.” HF4, Uganda  *Nurses torn between daily reality and formal procedures*  “And we have observed delays in care where you find there is **only a nurse, there is no doctor, but the procedure is before sending to ward there should be a clinician to first review the patient**. So we have observed, we have followed a patient journey where it was a neonate was brought to the emergency room, but because there was no clinician, there was only a nurse, so the nurse had a **conflicting loyalty** of should I refer this patient to the ward, should I attend to this patient, or call the clinician.” FGD, HF11, Uganda.  *Nurse-led care*  “Neonatal intensive care unit accommodates children < 30 days. It is **majorly run by nurses**. The in charge of the ward is a nursing officer (with a degree in nursing).” HF9, Uganda.  “Whenever a doctor is needed to review a patient at the Neonatal Intensive Care, the nurses on duty or midwife on duty makes a phone call to any of the doctors on duty attending to other wards (Maternity ward, Emergency department, Paediatric Inpatient Department, male and female wards). Whichever doctor available and free to help comes in, however most of the time, sick neonates are only managed by the nurses and midwives.” HF1, Uganda  *Unavailability of doctors*  “At times, there is one medical officer in the facility who is doing OPD and is called to IPD just for emergencies. He/she may not show up in time to intervene.” HF4, Uganda  “The medical doctor rarely comes to ward unless there is a specific patient he has come to review.” HF8, Uganda  “During second visit to the facility, the association of consultant doctors…and association of resident doctors were all in strike. Therefore, only medical officers and some house officers are running the clinic and the clinic is only general clinic with no specialty.” HF25, Nigeria  “At the emergency ward, there is no assigned doctor. Any doctor who is on call works for straight one week and covers the whole facility. This is as a result of excessive shortage of staffs.” HF13, Nigeria  “The nurse on duty reported that she did not know how to calculate dosages of antibacterials, anti-convulsants and IV fluids. The nurse had to constantly call the medical doctor and colleagues for guidance who did not respond to the call.” HF1, Uganda. |
| Most experienced staff reserved for inpatient wards | “During earlier visits, both nurses and CHEWs were observed at the triage area, but currently, only CHEWs are present. It was explained that the scarcity of nurses has necessitated their redeployment to the wards.” HF22, Nigeria. |
| Access to emergency medicines | *Responsibility of caregivers*  “At the pharmacy, the prescribed medication is costed, and the caregiver makes payment at the pay point. After payment, a receipt is given to the pharmacy and prescribed medication is dispensed and given to the caregivers alongside the admission pack. Caregivers return to the emergency unit with the purchased medication. Health workers continue with the treatment.” HF20, Nigeria.  “Like for [HF21], if they prescribe medication, they just **give the prescription form to the caregiver and ask him to go to pharmacy and purchase the drug**. But if they have available ones in their cupboard, for example, emergency pediatric, they have large cupboards there where they keep some emergency drugs in their consumables.” FGD, HF21, Nigeria.  “If drugs are prescribed for the patient, it's **left for the patient to decide where they will purchase the drug, either at the facility pharmacy or outside pharmacy** but on observation, most times, the pharmacy doesn't have the prescribed drugs and the patients are left with no other option but to go outside the facility to get it.” HF13, Nigeria.  “There is frequent stock-out of drugs and medications at the store department. The patients or their caregivers are required to buy these essential supplies from external pharmacies, which negatively impacts the timely provision of treatment, especially for paediatric emergencies.” HF2, Uganda.  *Community adaptation*  “At the emergency trolley, there was no diazepam. So they're, like, asking the matron, ah, why didn't you order for, you know, this thing. Like, the child was having severe seizures there for long. No diazepam. They were just confused, running up and down. **Then a caregiver [of another child] that was watching the whole thing… was now like, doctor, come and take diazepam for me.** I have. When you people have, you can replace it.” FGD, HF26, Nigeria.  *Multiple steps from prescription to access*  “**There are no emergency drugs kept in the emergency tray** at the paediatric ward because drugs are requisitioned after prescription by the medical doctor or medical clinical officer. It is time-consuming as the requested drugs need to first be approved by the office of the matron before the pharmacy can issue the drugs to the enrolled nurse on duty to administer to the patients.” HF7, Uganda.  “There is frequent stock-out of drugs and medications at the store department. The patients or their caregivers are required to buy these essential supplies from external pharmacies.” HF2, Uganda. |
| Informal referrals | “In the event that the ward is filled up with patients, children are attended outside the ward, **those that need referral, they are just told to go to another facility due to lack of bed space**. They **don't usually give them referral letter** rather they just tell them verbally.” HF15, Nigeria. |
| Administration steps | *Delaying or complicating clinical assessment*  “Every patient coming to the facility for the first time must first do a retroviral screening to know their HIV status after which, payment at the paypoint is done for registration and folder opening , receipts are been issued to the patients by the cashier, then the patient goes to the medical record department with the receipt to tender as evidence of payment.” HF13, Nigeria  “On getting to the record department, they will be given a form to fill. She will then instruct them to go downstairs to meet with cashier at the children emergency department to pay for opening of folder and consultation. A printout receipt for payment will be given to them as evidence of payment which they take back upstairs to tender to the record officer at the children record department. The record officer will them fill in the biodata of the child on a new folder from the form which the caregiver previously filled. The record officer will also give them a small hospital visiting card containing the child's name and folder number which they are to be bringing to the hospital whenever they want to access care. After which, she will ask them to go to the waiting area to sit while she takes the folder to the nurse at the nurses’ station…There is no rigid policy of payment before treatment in emergency situation.” HF26, Nigeria  “At [this facility], whether critically ill or not, patients first proceed to the record office, which doubles as the card collection point and is located about 2–3 minutes’ walk from the main gate.” HF17, Nigeria  “The caregiver was directed from post-natal ward to SCBU (Special Care Baby Unit) and she asked a sub-staff [cleaners] on how to see the doctor. The sub-staff around asked the woman to go to the record office and buy [an] OPD card. The caregiver then came back after buying [the] OPD card and join[ed] the queue.” HF22, Nigeria.  *Expedited or in parallel with clinical assessment*  “As the child is being worked on, one of the triage health workers comes and collects the child’s biodata (name, age, sex, next of kin, place of birth) from the caregiver. This is done later once consent is obtained from the caregiver to prevent wasting time since the child needs quick attention.” HF3, Uganda  “As the child is being assessed, the care giver is sent to registration window for registration.” HF5, Uganda  For paediatric emergency unit, during our first visit, the record personnel share tables with nurses at the nurses station within the emergency/clinic due to lack of space, this is to help caregivers to activate/open new cards without stress delay.” HF16, Nigeria |

## Table I. Characteristics of researchers participating in focus group discussions in Uganda and Nigeria

|  | **Uganda (n=7)** | **Nigeria (n=9)** | **Overall (n=16)** |
| --- | --- | --- | --- |
| **Median (interquartile range) age years** | 28 (27-33) | 33 (31-36) | 32 (28-35) |
| **Gender n (%)** | Female: 6 (86%)  Male: 1 (14%) | Female: 7 (78%)  Male: 2 (22%) | 13 (81%)  3 (19%) |
| **Professional background** | Pharmacist: 3 (43%)  Nurse: 2 (29%)  Clinical officer: 2 (29%) | Nurse: 6 (67%)  Midwife: 1 (11%)  Public health: 1 (11%)  Doctor: 1 (11%) | 9 (56%)  3 (19%)  3 (19%)  1 (6%) |
| **Median (interquartile range) clinical experience years** | 4 (2.5-5.5) | 8 (5-11) | 5.5 (4-8) |
| **Median (interquartile range) research experience years** | 3 (3-4) | 5 (3-5) | 4 (3-5) |

**A**

**
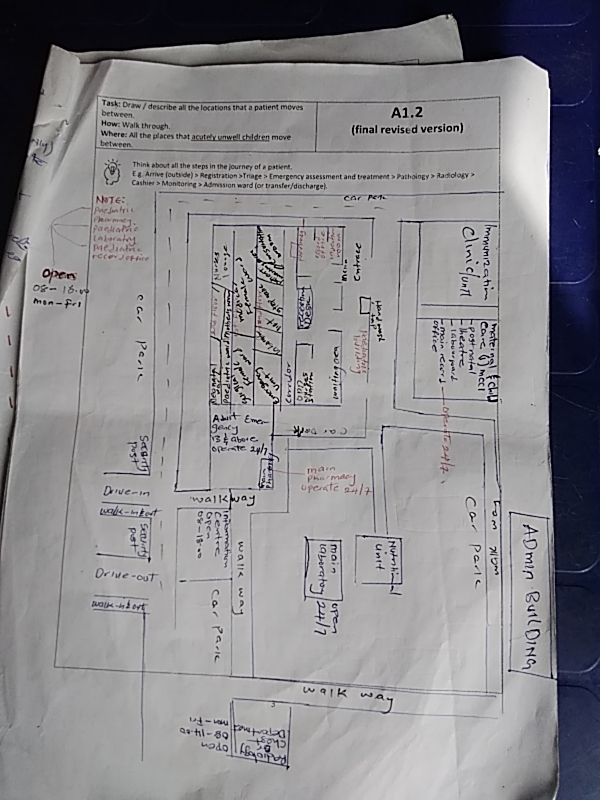
**

**B**

**
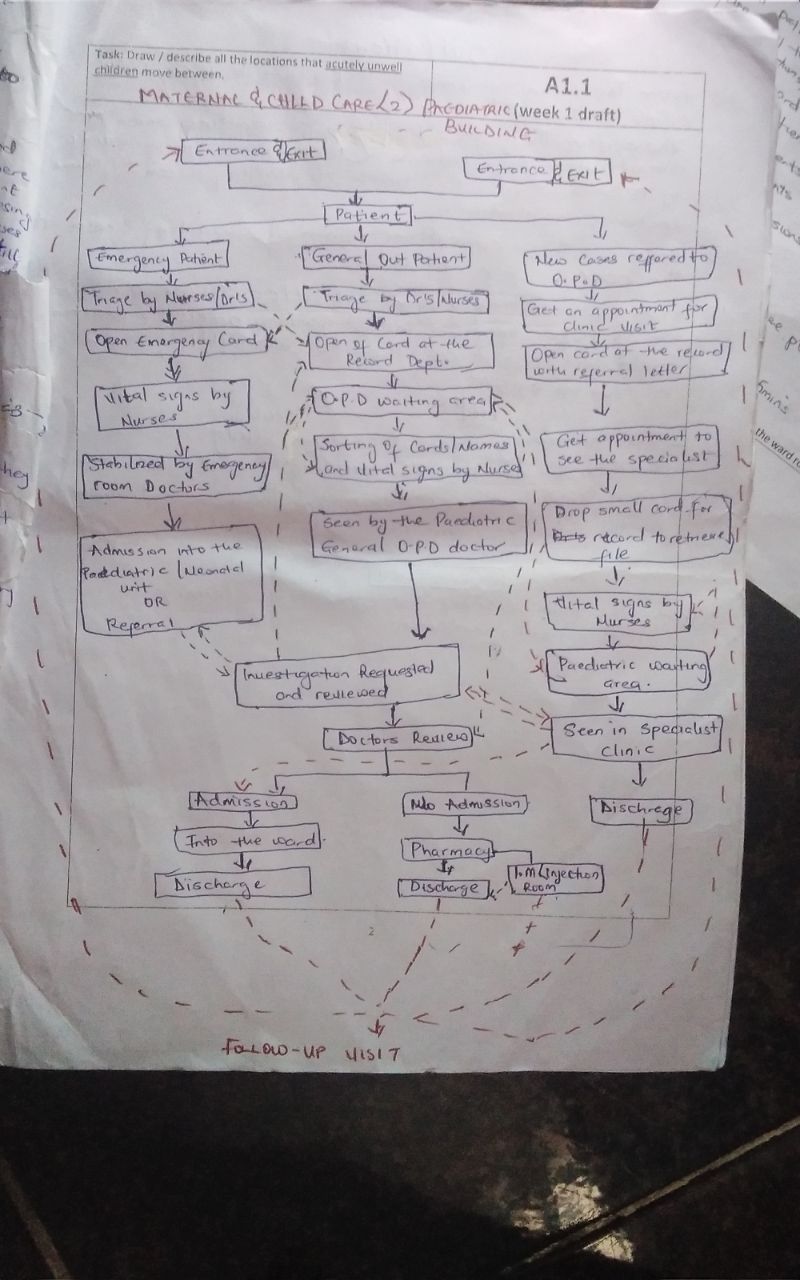
**

## Figure A. Example of the evolution of process maps over time, from simple spatial maps of facility organisation (A) to conceptual maps of processes (B). Maps were supplemented by narrative descriptions.

## Text A. Researcher positionality

A Doctor, nurse, and pharmacist team members were insiders to the clinical world (RS, MB, HG), including those working in Nigerian (AAB, AGF) and Ugandan (DM, FEK and FS) hospital contexts, bringing different perspectives on clinical practices, priorities, and workflows. This was complemented by the broader public health perspectives of non-clinician team members (AS, CK), qualitative research expertise (BM, FS, SK) and health policy expertise (FS and SK). Nigeria and Ugandan research supervisors and senior researchers (DM, AS, AAB, FK, FS and AGF) were both insiders and outsiders to cultural, linguistic, and health-system contexts depending on the study region. Facility-embedded research assistants (majority nurses and female) were purposively selected as insiders to the local setting, many with existing personal and professional relationships with facility staff.

## Text B. Process mapping data collection tool

We set out the following phases of process mapping, informed by the Antonacci et. al framework:^24^ 1- identify stakeholders; 2- hold informal conversations; 3- draw and narratively describe the facility organisation, resources and processes; 4- seek clarifications and revise the process maps iteratively.

Training in process mapping methodology was conducted on commencement, comprising a two-week program facilitated by clinicians and mixed methods researchers. A refresher training was conducted half-way through data collection in order to further enhance data quality, in addition to weekly supervision and reflection meetings by research supervisors in each country. We brought research assistants together for in-person team meetings at the project’s mid-point (6 months) and endpoint (12 months).

|  | | **A1.1**  **(week 1 draft)** | |
| --- | --- | --- | --- |
| Your name: | |  |  |
| Record ID (from REDCap): | |  |  |
| Facility name: | | | |
| **Task:** Draw / describe all the locations that a patient moves between.  **How:** Walk through.  **Where:** All the places that acutely unwell children move between.  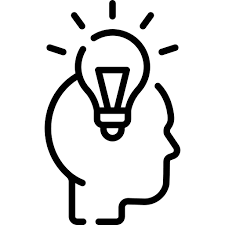Think about all the steps in the journey of a patient.  E.g. Arrive (outside) > Registration >Triage > Emergency assessment and treatment > Pathology > Radiology > Cashier > Monitoring > Admission ward (or transfer/discharge). | | | |
| **Task:** Draw / describe all the locations that acutely unwell children move between. | | **A1.1**  **(week 1 draft)** | |
|  | | | |
| **Task:** Draw / describe all the locations that a patient moves between.  **How:** Walk through.  **Where:** All the places that acutely unwell children move between. | | **A1.2**  **(final revised version)** | |
| 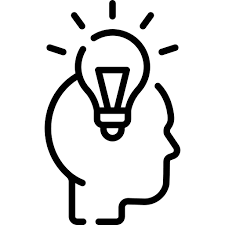Think about all the steps in the journey of a patient.  E.g. Arrive (outside) > Registration >Triage > Emergency assessment and treatment > Pathology > Radiology > Cashier > Monitoring > Admission ward (or transfer/discharge). | | | |
| **Task:** Draw / describe all the locations that acutely unwell children move between. | | **A1.2**  **(final revised version)** | |
|  | | | |
| **Task:** Absorb, understand and describe/draw care processes for children.  **How:** Sit, watch and chat in each location.  **Where:** Locations where children are first assessed  (e.g. emergency/outpatients). | | **B1.1**  (first assessed)  Location 1: __________________ | |
| **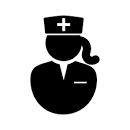**  **Focus on healthcare workers.**  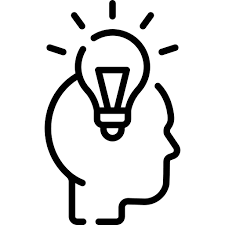  Think about number of staff, their roles (e.g. nurses, doctors, other), how they move between locations, and what happens after-hours. | | | |
| **Task:** Absorb, understand and describe/draw care processes for children.  **How:** Sit, watch and chat in each location.  **Where:** Locations where children are first assessed  (e.g. emergency/outpatients). | | **B1.1**  (first assessed)  Location 1: __________________ | |
| **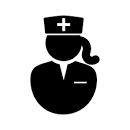**   - During a weekday working hours   - Number of Nurses/Midwives: _____   - Number of Doctors (qualified): _____   - Other staff involved in care (please write their role and number):   _________________________­­­ _________________________­­­ ___________________________­­­   - During a weekday evening   - Number of Nurses/Midwives: _____   - Number of Doctors (qualified): _____   - Other staff involved in care (please write their role and number):   _________________________­­­ _________________________­­­ ___________________________­­   - During a weekday overnight   - Number of Nurses/Midwives: _____   - Number of Doctors (qualified): _____   - Other staff involved in care (please write their role and number): ­­­   _________________________­­­ _________________________­­­ ___________________________   - During a weekend day   - Number of Nurses/Midwives: _____   - Number of Doctors (qualified): _____   - Other staff involved in care (please write their role and number):   _________________________­­­ _________________________­­­ ___________________________   - During a weekend overnight   - Number of Nurses/Midwives: _____   - Number of Doctors (qualified): _____   - Other staff involved in care (please write their role and number):   _________________________­­­ _________________________­­­ ___________________________ | | | |
| 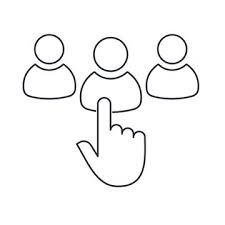**Focus on systems.**  **For the location** where children are first assessed (e.g. emergency/outpatients), describe the **process for staff to identify sicker patients:**  **Do staff have a formal process for emergency triage and assessment?** (e.g. colour-coded triage, ETAT, etc.)  □ No □ Yes | | | |
| **Task:** Absorb, understand and describe/draw care processes for children.  **How:** Sit, watch and chat in each location.  **Where:** Locations where children are first assessed  (e.g. emergency/outpatients). | | **B1.2**  (first assessed)  Location 1: __________________ | |
| 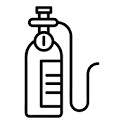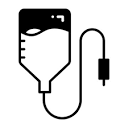  **Focus on medical equipment/devices and clinical guidelines.**  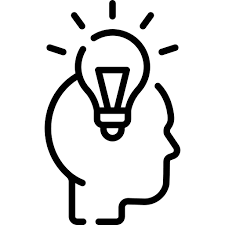  Think about what equipment you see, how many are available, whether they are functional, and how they move between locations. (E.g. oximeter, oxygen equipment and supplies, resuscitation equipment, clinical guidelines etc.) Think about who is using it, when, where and how. | | | |
| **Task:** Absorb, understand and describe/draw care processes for children.  **How:** Sit, watch and chat in each location.  **Where:** Locations where children are admitted  (e.g. children’s ward). | | **B2.1**  (admitted)  Location 2: __________________ | |
| **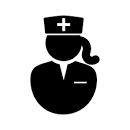**  **Focus on healthcare workers.**  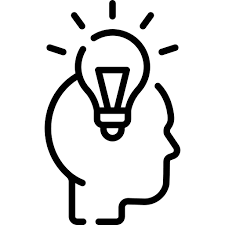  Think about number of staff, their roles (e.g. nurses, doctors, other), how they move between locations, and what happens after-hours. | | | |
| **Task:** Absorb, understand and describe/draw care processes for children.  **How:** Sit, watch and chat in each location.  **Where:** Locations where children are admitted  (e.g. children’s ward). | | **B2.1**  (admitted)  Location 2: __________________ | |
| **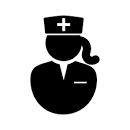**   - During a weekday working hours   - Number of Nurses/Midwives: _____   - Number of Doctors (qualified): _____   - Other staff involved in care (please write their role and number):   _________________________­­­ _________________________­­­ ___________________________­­­   - During a weekday evening   - Number of Nurses/Midwives: _____   - Number of Doctors (qualified): _____   - Other staff involved in care (please write their role and number):   _________________________­­­ _________________________­­­ ___________________________­­   - During a weekday overnight   - Number of Nurses/Midwives: _____   - Number of Doctors (qualified): _____   - Other staff involved in care (please write their role and number): ­­­   _________________________­­­ _________________________­­­ ___________________________   - During a weekend day   - Number of Nurses/Midwives: _____   - Number of Doctors (qualified): _____   - Other staff involved in care (please write their role and number):   _________________________­­­ _________________________­­­ ___________________________   - During a weekend overnight   - Number of Nurses/Midwives: _____   - Number of Doctors (qualified): _____   - Other staff involved in care (please write their role and number):   _________________________­­­ _________________________­­­ ___________________________ | | | |
| **Task:** Absorb, understand and describe/draw care processes for children.  **How:** Sit, watch and chat in each location.  **Where:** Locations where children are admitted  (e.g. children’s ward). | | **B2.2**  (admitted)  Location 2: __________________ | |
| 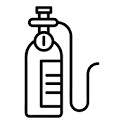  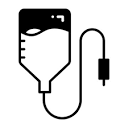**Focus on medical equipment/devices.**  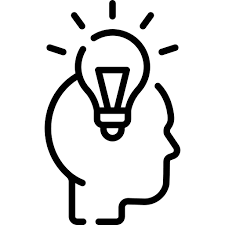  Think about what equipment you see, how many are available, whether they are functional, and how they move between locations. (E.g. oximeter, oxygen equipment and supplies, resuscitation equipment, clinical guidelines etc.) Think about who is using it, when, where and how. | | | |
| **Task:** Absorb, understand and describe/draw care processes for children.  **How:** Sit, watch and chat in each location.  **Where:** Additional locations where children are first assessed or admitted (optional). | | **B3.1**  (other location – optional)  Location 3: __________________ | |
| **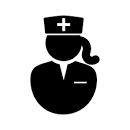**  **Focus on healthcare workers.**  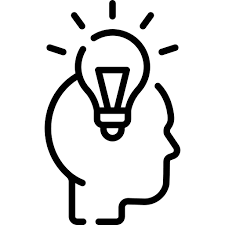  Think about number of staff, their roles (e.g. nurses, doctors, other), how they move between locations, and what happens after-hours. | | | |
| **Task:** Absorb, understand and describe/draw care processes for children.  **How:** Sit, watch and chat in each location.  **Where:** Additional locations where children are first assessed or admitted (optional). | | **B3.1**  (other location – optional)  Location 3: __________________ | |
| **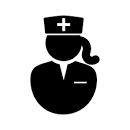**   - During a weekday working hours   - Number of Nurses/Midwives: _____   - Number of Doctors (qualified): _____   - Other staff involved in care (please write their role and number):   _________________________­­­ _________________________­­­ ___________________________­­­   - During a weekday evening   - Number of Nurses/Midwives: _____   - Number of Doctors (qualified): _____   - Other staff involved in care (please write their role and number):   _________________________­­­ _________________________­­­ ___________________________­­   - During a weekday overnight   - Number of Nurses/Midwives: _____   - Number of Doctors (qualified): _____   - Other staff involved in care (please write their role and number): ­­­   _________________________­­­ _________________________­­­ ___________________________   - During a weekend day   - Number of Nurses/Midwives: _____   - Number of Doctors (qualified): _____   - Other staff involved in care (please write their role and number):   _________________________­­­ _________________________­­­ ___________________________   - During a weekend overnight   - Number of Nurses/Midwives: _____   - Number of Doctors (qualified): _____   - Other staff involved in care (please write their role and number):   _________________________­­­ _________________________­­­ ___________________________ | | | |
| **Task:** Absorb, understand and describe/draw care processes for children.  **How:** Sit, watch and chat in each location.  **Where:** Other locations where children are first assessed or admitted (optional). | | **B3.2**  (other location – optional)  Location 3: __________________ | |
| 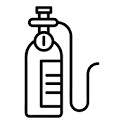  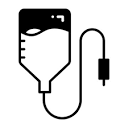**Focus on medical equipment/devices.**  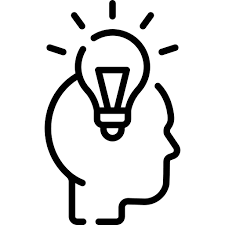  Think about what equipment you see, how many are available, whether they are functional, and how they move between locations. (E.g. oximeter, oxygen equipment and supplies, resuscitation equipment, clinical guidelines etc.) Think about who is using it, when, where and how. | | | |
| **Task:** Understand and describe/draw care processes for children with specific conditions.  **How:** Sit, watch and chat in each location.  **Where:** Do one description/drawing for the journey between the locations described in section B. | | **C1** | |
| 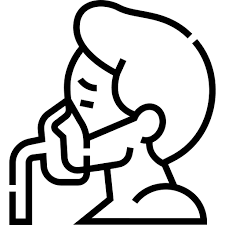**Focus on the child with severe difficulty breathing:**  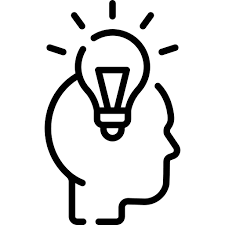  Think about where, what, who, how, why in all steps. | | | |
| **Task:** Understand and describe/draw care processes for children with specific conditions.  **How:** Sit, watch and chat in each location.  **Where:** Do the one description/drawing for the journey between the locations described in section B. | | **C2** | |
| 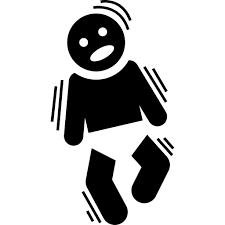**Focus on the child with coma or convulsions:**  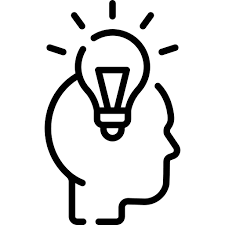  Think about where, what, who, how, why in all steps. | | | |
| **Task:** Understand and describe/draw care processes for children with specific conditions.  **How:** Sit, watch and chat in each location.  **Where:** Do the one description/drawing for the journey between the locations described in section B. | | **C3** | |
| 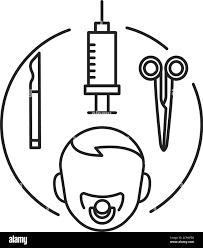**Focus on the child with a surgical condition:**  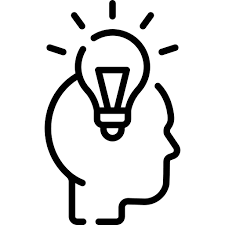  Think about where, what, who, how, why in all steps. | | | |
| **Task:** Understand and describe/draw care processes for children with specific conditions.  **How:** Sit, watch and chat in each location.  **Where:** Do the one description/drawing for the journey between the locations described in section B. | | **C4** | |
| 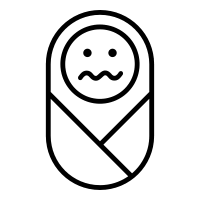**Focus on the sick neonate (<28 days old):**  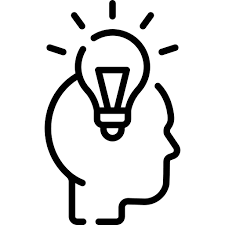  Think about where, what, who, how, why in all steps. Consider inborn and outborn. | | | |
| **Task:** Describe factors that enable children to receive the care they need.  **How:** Observation and conversation.  **Where:** Overall impressions for all locations, and/or for each location described in section B. | | **D1** | |
| 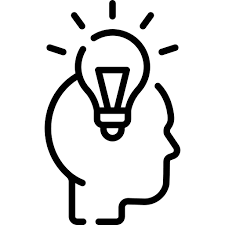  Think about structures, systems, practices, resources. | | | |
| **Task:** Describe factors that may make it more difficult for children to receive the care they need.  **How:** Observation and conversation.  **Where:** Overall impressions for all locations, and/or for each location described in section B. | | **D2** | |
| 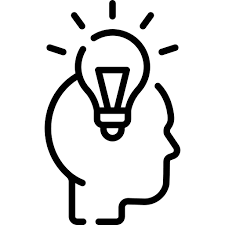  Think about structures, systems, practices, resources. | | | |
| **Task:** Document who you spoke with to get the information in this form. | | **E** | |
| Name of the person | Role of the person(s) | | Date of discussion |
| **Other comments / observations / reflections?** | | | |

## Text C. Focus group discussion guide

Provide information sheet, and consent forms

**Welcome:** Thank you for joining us today. The aim is to have the opportunity to learn from your personal experiences in conducting REAL-MOXY research.

**Overview:** You have been involved in REAL-MOXY now for more than 12 months. We set out to learn how oxygen and pulse oximetry are used in facilities, and to understand how unwell children receive care more broadly.

We know that you have spent many hours following patients, interacting with staff, learning and observing different ways that things are done in different hospitals. And you’ve documented these very well in the process maps and patient journeys. Today, we want to make sure that we learn as much as possible from your expertise, especially the things that may have been difficult to document.

There are no right or wrong answers. As much as possible, we want you to think about concrete examples of what you have observed: the good, the bad, the difficult challenges and the clever solutions. You can tell us the facility you are referring to in examples. We don’t need to know the names of staff or patients.

We are also interested in learning about your experience in using REAL-MOXY methods – this has been a challenging study, with unique approaches. We want to make sure we learn from your reflections.

**Recording:** We will be recording this session to make sure we capture everything you tell us. We will use you names during our discussion, but will not quote your names in reports or publications. Please keep the details of today’s discussion confidential outside this room.

Do you have any questions?

**We will now start recording **

**Questions – content**

| **Question 1**  We have noticed some facilities have systems for triaging sick children (e.g. ETAT).  Can you tell us how you saw healthcare workers use or not use these systems to identify and manage the sickest children? | Can you give me examples?  When did these systems work well / not well? |
| --- | --- |
| **Question 2**  We have noticed some facilities do not have a known system for triaging sick children.  Can you tell us how you saw healthcare workers identify and manage the sickest children? | Can you give me examples?  What approaches worked / did not work? |
| **Question 3**  We noticed from patient journeys that healthcare workers can be pulled in many directions – sorting through very sick patients, well patients, currently admitted patients. How do they go about priortising what their most important task is? | Can you give me examples?  Positive and negative  Nurses / doctors / clinical officers |
| **Question 4**  CHAI’s work has focussed on pulse oximetry and oxygen. How did you see pulse oximetry fit in with how healthcare workers prioritise very sick children? | Helpful / unhelpful  Nurses / doctors / clinical officers  Relationship with antibiotics, fluids, IV access.  Can you give me examples? |
| **Question 5**  How do health care workers think about oxygen alongside other therapies? | Think about fluids, antibiotics  Can you give me examples? |
| **Question 6**  We have noticed that parents/caregivers play an important role, and are often part of the treating team. Was this your experience? | Can you give me examples?  When does this work well / not worked?  Positives / negatives |
| **Question 7**  We have noticed that auxiliary staff (e.g. gatekeepers) and community health care workers have an important role in triage and emergency care. Was this your experience? | Can you give me examples?  When does this work well / not worked?  Different cadres  Positives / negatives |
| **Question 8**  We want to hear from each of you imagining you have a sick child that you need to take to hospital. Based on all you’ve learned what would you like to see done better? | How?  Barriers? |
| **Question 9**  At the start we said we wanted to have this discussion group so that we could learn from the experts – from you.  What has it been like being part of this Discussion Group? |  |

## Text D. Consent – procedures and resources

Informed Consent is a voluntary agreement to participate in research. It is important that participants in research studies understand what the research is about, what taking part means for them, and what will be done with the information they provide. We cannot collect information or data from research participants without first asking for their permission. Informed consent has three steps:

1. Provide the participant with study information.

2. Allow the participant to ask questions: It is very important that the participant can ask questions, both about the study overall, but also about their involvement. Answer these honestly and check for understanding.

3. Confirm their consent: Once they have been given information and time to ask questions, ask participants to sign the consent form. Offer the participant a copy of the information sheet to take with them.

Providing information

It is important that the participants understand the reason for conducting the interviews / discussions, your role, what types of questions will be asked, and how the information collected will be used.

- Introduce yourself, the research team and the research

- Explain the different research components – patient journeys, interviews, clinical care forms…

- Explain the interview – duration 45 mins, confidential and deidentified, recorded

- Consent

- Start recording

Suggested scripts

A) For healthcare worker interviews, managers and technician interviews:

“My name is XX. I am from XX, and have trained as a XX. I am part of a large research team, working in multiple countries including Nigeria/Uganda. We have learned and heard that at times there are barriers in the way to delivering the sort of care you want to unwell children (*or for technicians/managers, there are barriers to you doing your job effectively). And there are also barriers in the way for these children and families to access the care they need. We want to help clinicians, health facilities, and policy makers to understand these barriers. We also want to celebrate the things that are currently going well. And through these learnings, develop lessons and strategies that can benefit your facility, and many other facilities internationally.

One important life-saving therapy is oxygen. Oxygen is unique because it relies on many aspects of the health system working together (e.g. healthcare workers with biomedical technicians). And so by understanding it, we can get insights into the system more broadly.

You would have seen our data collectors following the journey of patients, observing clinical care on the wards, and collecting data from the medical records. In this part of the research we are asking you to participate in interviews/discussions. We will aim to put all this information together to develop a rich picture of oxygen services, and therefore the broader system of how unwell children receive care.

We will ask you mostly questions about your experiences, perspectives, and views. There are no right and wrong answers. We are interested in hearing as much as possible from you.

We will be taking notes as you speak. We will also be recording our conversation so we can spend time closely learning from what you have said. The interview will be transcribed (written down). From your interview, alongside that of many others, we will aim to draw out important lessons. We will not identify you by name. When referencing what you say, we will mention your position (e.g. nurse, doctor, administrator) and the country you are from (but not the name of the health facility). The interview will take between 45mins to 1 hour.

We will also collect some basic information about you, so that we can understand your background. This information will be reported generally (e.g. nurse, Uganda, 32 yo), and your name will be deidentified.”

If at any stage you feel uncomfortable, please tell me.

Do you have any questions?

Could you please sign this form to say that you understand the research and are happy to take part in it.”

### Staff Information and Consent Form

Project Name: Realist Evaluation and Learning in a Multi-country Medical OXYgen Program (REAL-MOXY)

Project Number: 97817

Short Name of Project: REAL-MOXY

Coordinating Principal Investigator: A/Prof Hamish Graham

Local Principal Investigator: XX

Version Number: 1 Version Date: 11/05/2023

We are inviting you to take part in an interview/focus group discussion about oxygen management for children and neonates.

This letter gives you more information about the interview. Taking part in the interview is up to you. You can say no if you want to.

What the project is about

We are conducting research on how oxygen and pulse oximetry are used in the care of unwell children. The study is run by MCRI and [local academic institute] XX across 10 facilities.

We aim to understand the enablers and barriers in effective oxygen therapy. We are asking you to take part because you have responsibility or involvement in wards caring for children.

We are conducting in-depth interviews and focus group discussions to understand:

• How patients are assessed, triaged, and managed from presentation to admission or discharge

• How pulse oximetry is used in decision making

• How oxygen is prescribed, commenced, adjusted and stopped

• Patient flow through your facility

• Barriers and enablers to effective oxygen therapy

Understanding these aspects will help us find solutions so more patients receive oxygen when they need it.

1. Who is running the project?

This project is being run by [insert country specific institutions] in collaboration with the Murdoch Children’s Research Institute (MCRI) Australia and the University of Melbourne, Australia.

It is taking place in 10 facilities in 6 countries (Uganda, Nigeria, Lao PDR, Cambodia, Rwanda, Liberia).

3. Why are we asking you to take part?

We are asking you to take part in this study because you are part of a team caring for children or supporting wards that care for children.

4. What do you need to do in this research project?

We will ask you questions about your day-day practice, and about how you make clinical decisions. Interviews will be initially between you and a research assistant. We may ask you to also participate in focus group discussions. Interviews and focus group discussions will last between 1-1.5 hours. Interviews will be recorded and transcribed.

We will provide refreshments, and re-imburse you for any costs you incur by taking part in the research.

Interviews will be recorded and transcribed. We will deidentify the recording and transcript, and assign it a research code. A spreadsheet linking this code with your identifying details will be kept securely by the principal investigator, and destroyed within 12 months of completion of the research activities.

5. Can you withdraw from the project?

Yes. You can stop taking part in the project at any time – even after the interview is completed. You just need to tell us. You do not need to tell us the reason why.

6. What are the possible benefits for you and other people in the future?

You will have the opportunity to share your experience with medical oxygen and pulse oximetry. While there are no direct benefits to you from participation, our aim is to learn how to better provide medical oxygen services to patients both in [COUNTRY] and in other countries facing similar challenges. Our reports will be made publicly available.

7. What are the possible risks, side effects, and inconveniences?

There are no risks or side-effects to being involved in this study because we are not providing any new or different treatments. We will not provide you with payment to take part of the study. But we will provide you with small refreshments.

8. How will we keep your information confidential?

We will collect and use general professional information about you for research purposes. We will store your electronic information securely on a research server and keep paper copies of our interview notes in a secure research office. Only research staff have access to this information. When we share the results of the research, we will first remove all personal identifiers, and identifiers of your workplace. All information identifying you will be destroyed within 12 months of completing the study. Non-identifying results will be kept for 10 years (or as required by local jurisdiction).

9. Who should you contact for more information?

You can choose to withdraw from the research at any stage. If you do so, we will delete all recordings / transcripts and destroy paper records.

Deidentified results will be used in reports and publications. Data used in analysis and reporting will be deidentified.

If you have any questions about the project, you can contact [insert name of researcher] on [insert phone number]. You can also email them at [insert email address].

This research was approved by [add local ethics committee details and contact].

Thank you very much for your time.

Yours sincerely

<Insert name>

<Insert position>

<Insert department and institution>

### Consent Form

Project Name: Realist Evaluation and Learning in a Multi-country Medical OXYgen Program (REAL-MOXY)

Project Number: 97817

Short Name of Project: REAL-MOXY

Principal Investigator: A/Prof Hamish Graham

Version Number: 1 Version Date: 11/05/2023

• I have read this information statement and I understand its contents.

• I understand what I have to do to be involved in this project.

• I understand the risks I could face because of my involvement in this project.

• I voluntarily consent to take part in this research project.

• I have had an opportunity to ask questions about the project and I am satisfied with the answers I have received.

• I understand that this project has been approved by The Royal Children’s Hospital Melbourne Human Research Ethics Committee, and [Local ethics board].

• I understand I will receive a copy of this Information Statement and Consent Form.

Participant Name Participant Signature Date

Declaration by researcher: I have explained the project to the participant who has signed above. I believe that they understand the purpose, extent and possible risks of their involvement in this project.

Research Team Member Name Research Team Member Signature Date
