# Supplementary material for: Pathways of emergency care for severely ill children in Nigerian and Ugandan hospitals: A process mapping study
Source: PLoS Med. 2026 May 6;23(5):e1004832. doi: 10.1371/journal.pmed.1004832 (PMC13166908; doi:10.1371/journal.pmed.1004832)
Supplement: Attachment — Submitted filename: Response to reviewers.docx [file pmed.1004832.s003.docx]

16^th^ February 2026

Dear editor and reviewers,

Thank you to the editors and reviewers for such thoughtful feedback. We have addressed the reviewer points below.

**Comments from the reviewers:**

Reviewer #1: This sentence in the "Results" section is confusing and unclear: First contact and early sorting of children into these pathways frequently involved guards, lay bystanders, students, and caregivers, with duplicated assessments when assessment and treatment occurred in different locations and misrouting of patients away from intended pathways. Consider making it two sentences.

Thank you for this feedback. This has been revised for clarity:

*“Across sites, formal triage systems were either absent or rarely used. First contact and early sorting of children into these pathways frequently involved guards, lay bystanders, students, and caregivers. Duplication in assessment and treatment steps and misrouting from intended pathways occurred especially when initial care was spread across multiple locations.”* Abstract, Results, page 3.

The acronym "FGD" in Design/Methods is not previously defined.

Thank you. We have avoided the acronym in the abstract (page 3), and defined it in the main text (page 5).

The references are included twice - pages 22-25 and again on pages 67-69
Thank you, we have removed the reference list at the end of the appendices.


Reviewer #2: This is a well-conducted process mapping study on the pathways of emergency care for severely ill children in Nigerian and Ugandan hospitals. This is basically a qualitative, multi-method study based on FGDs, therefore not much of statistical aspects that I can comment on as a statistical reviewer. However, there are still a few issues needing attention.

1. It says in the abstract 'We conducted a qualitative, multi-method study, across 26 purposively selected secondary and tertiary facilities in Uganda and Nigeria between October 2023 to December 2024'. However, there is no explicit justification on the selection of these 26 facilities for analyses. How representative are these facilities for Nigerian and Ugandan hospitals as a whole?

Thank you for this comment. We have added some more detail to clarify the sampling of the program itself in the Methods, as well as the philosophy behind facility selection for this study.

*“The study was conducted as part of the evaluation of a multi-country Medical OXYgen program (MOXY) implemented by the Clinton Health Access Initiative (CHAI) in partnership with government, which aimed to improve medical oxygen services. Regions were identified by the MOXY program as high priority for intervention based on high clinical demand for oxygen and/or weak oxygen systems.*

***Our aim was not to select a nationally representative sample of hospitals in Nigeria or Uganda, but to understand diversity in facility contexts within the opportunity afforded by the program. Therefore, we included facilities of varying sizes and levels of resourcing, categorising them as small secondary (large health centres or general hospitals), large secondary (provincial hospitals or general hospitals with specialist services) and tertiary hospitals (highest level of care, in urban centres).*** *We purposively oversampled smaller, more remote facilities which are usually less represented in the emergency and critical care literature.* ***Most included facilities were government-run; a small number of privately run facilities were included in contexts where these were the main paediatric inpatient care provider to the surrounding community.”*** Methods, Context, page 5.

2. There are no explicit annotations for abbreviations such as FGDs and ICA. It is difficult to follow for readers who are not in the field.
Thank you, we have now defined all abbreviations on first use.

3. An expert with knowledge and experience of the process mapping tool is needed to thoroughly review the analyses results.
We appreciate this, and would welcome any further methodological feedback.


Reviewer #3: Thank you for the privilege of reviewing this manuscript. I would like to commend the authors for this work. Overall, this is a strong, thoughtful, and policy-relevant qualitative study that addresses a critical but under-described gap in paediatric emergency care in low-resource settings, that is, how care is actually organised and delivered in real-world facilities. The use of prolonged facility embedding, process mapping, and cross-country comparison is a major strength, and the proposed organizational "archetypes" provide a useful and transferable conceptual framework. The manuscript is well written and methodologically rigorous. I believe with some adjustments/revisions we could have a much improved paper.

We greatly appreciate these encouraging words, thank you.

1. The manuscript clearly positions itself alongside ETAT and other standardized/guideline-based emergency care approaches, but the added conceptual value of the archetype framework could be articulated more explicitly and earlier. While this is addressed well in the discussion, the introduction could more clearly state why existing frameworks are insufficient for understanding real-world organizational care and how archetypes complement, rather than compete with, guideline-based approaches.
A short paragraph explicitly stating what this study enables, whereas previous facility readiness assessments or intervention studies do not, would strengthen the framing.

We have added the following to the introduction:

*“One constraint in effective implementation of emergency care interventions at large-scale has been limited data and understanding on the diversity of contexts in which interventions are introduced, including the organisational structures of health facilities.* ***Standardised tools such as ETAT and related guidelines provide critical clinical direction on the recognition and treatment of severely ill children. However, they offer limited insight into how facilities, as organisations, are structured to receive, triage, and treat patients in practice, or how care pathways unfold across physical spaces, teams, and time. Facility readiness assessments similarly tend to focus on the presence of resources or adherence to protocols, rather than on how care is operationalised within complex health system environments.***

***In this study, we sought to describe recurring patterns of facility organisation and patient flow (‘archetypes’) to help inform program theories for future interventions to improve emergency care for children.”*** Introduction, page 5.

2. The four archetypes look intuitively appealing and seem well supported by data, but how stable are they over time, with evolving HR and logistical ecosystems in SSA? And are facilities expected to fit one archetype neatly, or are hybrids anticipated?
I suggest adding a brief clarification (perhaps in the discussion) that archetypes are heuristic tools rather than rigid categories and that facilities may shift between them depending on staffing, time of day, or resource availability. This would pre-empt over-interpretation and enhance transferability.

Thank you. We have added the following to the discussion:

*“These archetypes should be understood as heuristic tools to support process understanding, rather than as fixed or country-specific classifications. The same facility may shift between archetypes over time, or in response to day-to-day variation and specific circumstances.”* Discussion, page 12.

3. One of the most compelling findings is the central role played by guards, caregivers, students, and bystanders in triage and navigation (this is also my lived reality). Now, while this is well described, the implications are not fully unpacked. Questions that quickly come to mind are: To what extent should health systems formalise these roles? And where do the authors see the balance between legitimising informal adaptations and safeguarding quality and accountability?
Even a short reflective paragraph acknowledging these tensions would strengthen the manuscript and avoid the perception that the paper implicitly endorses all informal practices. (To avoid the pitfalls of task shifting as a policy)

We have added the following to the discussion:

*“Secondly, we observed that facilities develop organic adaptations that address local challenges, including task shifting and delegation. Where these adaptations are positive, they risk being inadvertently subverted if ‘standardised’ emergency care intervention packages are implemented without effective co-design or understanding of context.27-29*

*The role of task-shifting has been extensively documented, and it is often adopted informally as a pragmatic response to workforce shortages.30,31 In the emergency care context, published experience with lay-personnel triage,32,33 nurse-led emergency assessment and management,34,35 vital sign monitoring and response systems by volunteers,36 and family-assisted patient monitoring37 have demonstrated promising results.* ***At the same time, not all such adaptations are appropriate or desirable,*** *and concerns have been raised regarding compromised quality and safety of care,38 as well as excessive workload and risk of burnout among those to whom tasks are shifted.39* ***There remains a responsibility to safeguard quality, including recognising minimal standard of training and credentialling needed to deliver clinical functions safely.*** *Nonetheless our observations indicate that informal task-shifting is already occurring in day-day practice, but these practices are rarely reflected in formal policies and lack the legitimacy afforded by institutional recognition, regulatory frameworks, and leadership endorsement.30,40* ***This lack of visibility limits the ability of health systems to define boundaries, ensure accountability, and determine which clinical functions can be safely supported by non-clinical cadres.*** *Practically, our findings suggest that quality improvement efforts in emergency care should begin with a diagnostic phase that examines work as actually done rather than work as intended; that guidelines and mandates should acknowledge the gap between what is recommended and the capacity of those available to deliver it; and that governments should realign official scopes of practice with the realities of everyday clinical work,* ***while remaining grounded in clear standards of quality of care and patient safety.****”* Discussion, page 12.

4. While neonatal care pathways are discussed separately and somewhat briefly, are they intended as a secondary analytical strand or illustrative extensions of the archetype framework?
The authors can attempt to explicitly acknowledge the limits of neonatal data earlier in the Results section, rather than mainly in Methods/Limitations. If not possible, then a little more can be said in the discussion (limitations)

Thank you. We have added the following to the limitations:

*“As neonatal care pathways are closely intertwined with midwifery and maternity services, and were not the primary analytic focus, our descriptions of neonatal care are more brief and focus primarily on the locations of care rather than detailed processes.”* Discussion, limitations, page 13.

5. The description of inductive content analysis is clear, but the transition from codes to archetypes could benefit from a short, worked example. Consider a simple schematic or table in the main paper (not appendix) showing how observations led to archetype construction.

Thank you. We have now added Table 2 as an illustrative example.

6. While terms such as "OPD," "IPD," and "ED" are well defined (and maybe well understood by SSA readers), for a global readership, occasional reminders (especially early in the Results) would help.

Thank you. We have redefined these terms early in the results section.

Furthermore, please consider briefly defining "critical realist ontology" in plain language for non-qualitative specialists.

We have added the following explanation:

*“We used a qualitative, multi-method design informed by a critical realist ontology,14* ***in which reality exists independent of the observer, and can be understood by theorising the generative mechanisms that interact with context to bring about observable patterns of events and outcomes. The scope of this study was focused on describing the empirical findings (the what, who and how) to allow further explanatory realist enquiry (the why) in future work.***

*Our epistemology was pragmatic,15 in that our knowledge generation was directed at generating archetypes of common patterns of facility organisation in ways that are useful for intervention design and system improvement.”* Methods, Design, page 5.

7. While differences between countries are evident, the paper appropriately avoids over-comparison. However, one explicit statement clarifying that the archetypes cut across national boundaries (rather than being country-specific models) would be helpful.

We have added the following:

*“These archetypes should be understood as heuristic tools to support process understanding,* ***rather than as fixed or country-specific classifications****. The same facility may shift between archetypes over time, or in response to day-to-day variation and specific circumstances.”* Discussion, page 12.

8. The swimlane diagrams are excellent, but to me, they seem information-dense. Is it possible to have one simplified archetype diagram for the main text, with the others remaining in the appendix, to improve readability? For many, such an arrangement may be a relatively new graphical concept.

Thank you for this thoughtful comment. We acknowledge that the Swimlane diagrams are information-dense and may require careful reading at first glance. However, their purpose is to visually convey the complexity of emergency care processes across personnel, locations, and activities, even in facilities with seemingly simple configurations, and to illustrate the added value of process mapping as a systems tool. On balance, we believe retaining the diagrams in the main text strengthens the clarity of the archetypes and the overall contribution of the paper.

9. The conclusions are appropriate and measured. I believe one final sentence explicitly linking findings to national emergency care planning or hospital accreditation processes could enhance policy relevance.

We have added the following to the conclusion:

*“The findings have potential utility for national emergency care planning and hospital accreditation processes, particularly in conceptualising readiness beyond the presence of resources or guidelines alone.”* Conclusions.

Dr Arthur Kwizera


Reviewer #4: Comments on the manuscript "Pathways of emergency care for severely ill children in Nigerian and Ugandan hospitals: a process mapping study"

The multi-site study described processes in the delivery of emergency care in Nigeria and Uganda, two resource-limited countries with high child and neonatal mortalities. The study purposely selected smaller and more remote secondary and tertiary hospitals as these were underrepresented in literature. Using a mapping process and FGDs with researchers who were embedded in these facilities, the study revealed a variety of gaps - both inherent in the service delivery process and in the administrative aspect - that undermined the timely and efficient provision of emergency care. The findings highlight actionable areas that can help improve systems and eventually mitigate child deaths.

MY COMMENTS

The paper is well written and the main points are clearly articulated.
The methodology is rigorous and detailed in description.
The findings are robust and well documented.
The discussion is sufficient for the aim of the paper.

Thank you.

Nonetheless, there are areas that can still be improved.

The Abstract and the manuscript itself is quite lengthy. The language is not verbose but there are descriptions in the Methodology that can be edited to shorten the paper.

Thank you – we have shortened the methods section of the abstract (deletions in track changes).

We have reduced the word count in the following sections:

- Methods: Researcher characteristics, reflexivity and positionality
- Methods: Data processing and analysis
- Results: Removed one quote

The study facilities can do with more description aside from where they are and if they are secondary or tertiary hospitals. For instance, are these government or privately-run facilities? Are they under local government or national government supervision? Some of these data are in Table 1 but a line or two in this regard will help. Such information can help clarify the funding, staffing, and governance aspects later in the Discussion.

We have added the following:

“*Most included facilities were government-run; a small number of privately run facilities were included in contexts where these were the main paediatric inpatient care provider to the surrounding community.”* Methods, Context, p. 5

Similarly, it may not be enough to state that the researchers were "embedded" in the facilities. In studies with FGDs, a description of the study participants, even briefly (or even in the form of a table), is provided. The "Researcher Positionality" in the appendix does not suffice. For instance, how long have these researchers been working with their respective facility?

The embedded researchers were not employed by any of the participating facilities prior to this study.

This information will strengthen the reliability of their comments, especially if most of them have been with their facility for a long time.

Thank you, we have added some more information on the research assistant clinical and research experience to the table in the Appendix (p.30) and reference this in the positionality section.

The term "cost-cutting opportunities" in the Conclusion section should be reconsidered as it may be considered a misnomer. For one, cost-cutting measures are usually not regarded as opportunities. More importantly, in resource-poor or resource-limited settings, cost-cutting will only erode whatever is left that people are already hard-pressed to work with. Perhaps the term "cost-efficient measures" may be an alternative.

Corollary to the aforementioned point, the proposed "cost-cutting opportunities" in the Conclusion section should be fleshed out in a separate paragraph in the Discussion.

Thank you. We agree – cost-cutting is not what we intended to say, and we have removed this term. We have softened the language around cost of interventions, mindful that drawing conclusions about this falls outside the study’s scope.

Overall, this paper can be accepted for publication, but can still be improved with some editing and very minor revisions.
Thank you.

**Editorial requests**
Any attachments provided with reviews can be seen via the following link:
--------------------------------------------------------- ---
General editorial requests:
(Note: not all will apply to your paper, but please check each item carefully)

* We ask every co-author listed on the manuscript to fill in a contributing author statement, making sure to declare all competing interests. If any of the co-authors have not filled in the statement, we will remind them to do so when the paper is revised. If all statements are not completed in a timely fashion this could hold up the re-review process. If new competing interests are declared later in the revision process, this may also hold up the submission. Should there be a problem getting one of your co-authors to fill in a statement we will be in contact. Please do not add or remove authors without first discussing this with the handling editor. You can see our competing interests policy here: [http://journals.plos.org/plosmedicine/s/competing-interests](https://url.au.m.mimecastprotect.com/s/jyLkCOMKMASrQxRjspcQIG3RPk?domain=journals.plos.org).

Thank you. Please let me know if you have pending declarations.

* Please upload any figures associated with your paper as individual TIF or EPS files with 300dpi resolution at resubmission; please read our figure guidelines for more information on our requirements: [http://journals.plos.org/plosmedicine/s/figures](https://url.au.m.mimecastprotect.com/s/z2mICP7L7BcAVXGRfPfJIxfbzh?domain=journals.plos.org). While revising your submission, we strongly recommend that you use PLOS's NAAS tool ([https://ngplosjournals.pagemajik.ai/artanalysis](https://url.au.m.mimecastprotect.com/s/mIrJCQnMnDc4ZPApHYhLIGMBna?domain=ngplosjournals.pagemajik.ai)) to test your figure files. NAAS can convert your figure files to the TIFF file type and meet basic requirements (such as print size, resolution), or provide you with a report on issues that do not meet our requirements and that NAAS cannot fix.

After uploading your figures to PLOS's NAAS tool - [https://ngplosjournals.pagemajik.ai/artanalysis](https://url.au.m.mimecastprotect.com/s/mIrJCQnMnDc4ZPApHYhLIGMBna?domain=ngplosjournals.pagemajik.ai), NAAS will process the files provided and display the results in the "Uploaded Files" section of the page as the processing is complete.
If the uploaded figures meet our requirements (or NAAS is able to fix the files to meet our requirements), the figure will be marked as "fixed" above. If NAAS is unable to fix the files, a red "failed" label will appear above.
When NAAS has confirmed that the figure files meet our requirements, please download the file via the download option, and include these NAAS processed figure files when submitting your revised manuscript.
Done.

* Please ensure that the paper adheres to the PLOS Data Availability Policy (see [http://journals.plos.org/plosmedicine/s/data-availability](https://url.au.m.mimecastprotect.com/s/cheCCRONOESPZWm1sLivI1MFBW?domain=journals.plos.org)), which requires that all data underlying the study's findings be provided in a repository or as Supporting Information. For data residing with a third party, authors are required to provide instructions with contact information (web or email address) for obtaining the data. Please note that a study author cannot be the contact person for the data. PLOS journals do not allow statements supported by "data not shown" or "unpublished results." For such statements, authors must provide supporting data or cite public sources that include it.
The full facility process maps cannot be made publicly available because they contain detailed layout schematics that may permit identification of individual facilities, even after removal of explicit identifiers. De-identified facility summaries are provided in the Supporting Information files. Requests for access to additional materials may be directed to the relevant country institutional ethics committees (University College Hospital Ibadan / University College Hospital, Nigeria: uiuchec@gmail.com; Makerere University School of Health Sciences, Uganda: muwanguzinoreen@yahoo.com), which can review and, if appropriate, forward requests to facility leadership in accordance with local governance requirements.

* We expect all researchers with submissions to PLOS in which author-generated code underpins the findings in the manuscript to make all author-generated code available without restrictions upon publication of the work. In cases where code is central to the manuscript, we may require the code to be made available as a condition of publication. Authors are responsible for ensuring that the code is reusable and well documented. Please make any custom code available, either as part of your data deposition or as a supplementary file. Please add a sentence to your data availability statement regarding any code used in the study, e.g. "The code used in the analysis is available from Github [URL] and archived in Zenodo [DOI link]" Please review our guidelines at [https://journals.plos.org/plosmedicine/s/materials-software-and-code-sharing](https://url.au.m.mimecastprotect.com/s/o1AYCVARALhjQyw6sjswIEe6wN?domain=journals.plos.org) and ensure that your code is shared in a way that follows best practice and facilitates reproducibility and reuse. Because Github depositions can
be readily changed or deleted, we encourage you to make a permanent DOI'd copy (e.g. in Zenodo) and provide the URL.

The coding framework used for the analysis is provided in the appendices with example quotations.

FORMATTING - GENERAL
* Abstract: Please structure your abstract using the PLOS Medicine headings (Background, Methods and Findings, Conclusions). Please combine the Methods and Findings sections into one section.
Done.

* At this stage, we ask that you include a short, non-technical Author Summary of your research to make findings accessible to a wide audience that includes both scientists and non-scientists. The Author Summary should immediately follow the Abstract in your revised manuscript. This text is subject to editorial change and should be distinct from the scientific abstract. Ideally each sub-heading should contain 2-3 single sentence, concise bullet points containing the most salient points from your study. In the final bullet point of 'What Do These Findings Mean?', please include the main limitations of the study in non-technical language. Please see our author guidelines for more information: [https://journals.plos.org/plosmedicine/s/revising-your-manuscript#loc-author-summary](https://url.au.m.mimecastprotect.com/s/rcXoCWLVLMFwoq9Kilt6Io2hZo?domain=journals.plos.org).
Done.

* Please express the main results with 95% CIs as well as p values. When reporting p values please report as p<0.001 and where higher as the exact p value p=0.002, for example. Throughout, suggest reporting statistical information as follows to improve clarity for the reader "22% (95% CI [13%,28%]; p</=)". Please be sure to define all numerical values at first use.

N/A

* Please include page numbers and line numbers in the manuscript file. Use continuous line numbers (do not restart the numbering on each page).

Done.

* Please cite the reference numbers in square brackets. Citations should precede punctuation.

Done.

FIGURES AND TABLES
* Please provide titles and legends for all figures and tables (including those in Supporting Information files).

Done.

* Please define all abbreviations used in each figure/table (including those in Supporting Information files).
Done.

* Please consider avoiding the use of red and green in order to make your figure more accessible to those with color blindness.
Thank you. The use of colour in the images is in backgrounds only, and should not affect the clarity or interpretation for a reader with colour blindness.

SUPPLEMENTARY MATERIAL
* Please note that supplementary material will be posted as supplied by the authors. Therefore, please amend it according to the relevant comments outlined here.

* Please cite your Supporting Information as outlined here: [https://journals.plos.org/plosmedicine/s/supporting-information](https://url.au.m.mimecastprotect.com/s/26snCXLWLNF7rK5Rt2uKIWQV8I?domain=journals.plos.org)

REFERENCES
* PLOS uses the numbered citation (citation-sequence) method and first six authors, et al.

* Please ensure that journal name abbreviations match those found in the National Center for Biotechnology Information (NCBI) databases ([http://www.ncbi.nlm.nih.gov/nlmcatalog/journals](https://url.au.m.mimecastprotect.com/s/Ts_jCYW8WOToBl1qtlC9IxRtaZ?domain=ncbi.nlm.nih.gov)), and are appropriately formatted and capitalised.

* Where website addresses are cited, please include the complete URL and specify the date of access (e.g. [accessed: 12/06/2023]).

* Please also see [https://journals.plos.org/plosmedicine/s/submission-guidelines#loc-references](https://url.au.m.mimecastprotect.com/s/Si_dCZY1YPtnBg4LTkFrIB_dRq?domain=journals.plos.org) for further details on reference formatting.
Done.

QUALITATIVE STUDIES

* Please report your qualitative study according to the appropriate study design provided at ([http://www.equator-network.org/?post_type=eq_guidelines&eq_guidelines_study_design=qualitative-research&eq_guidelines_clinical_specialty=0&eq_guidelines_report_section=0&s=](https://url.au.m.mimecastprotect.com/s/VAd3C1WLWKTwWGzoiMHrIVQ708?domain=equator-network.org)) and provide the relevant completed checklist as a supplemental file. In the checklist, please include sufficient text excerpted from the manuscript to explain how you accomplished all applicable items. When completing checklists, please use section and paragraph numbers, rather than page numbers.

* We recommend that authors use the COREQ checklist, or other relevant checklists listed by the Equator Network, such as the SRQR, to ensure complete reporting (see: [http://www.equator-network.org/?post_type=eq_guidelines&eq_guidelines_study_design=qualitative-research&eq_guidelines_clinical_specialty=0&eq_guidelines_report_section=0&s=](https://url.au.m.mimecastprotect.com/s/VAd3C1WLWKTwWGzoiMHrIVQ708?domain=equator-network.org)). Please add the following statement, or similar, to the Methods: "This study is reported as per the Consolidated criteria for reporting qualitative research (COREQ): a 32-item checklist for interviews and focus groups (S1 Checklist)."

Done.

* In general, we expect qualitative studies to include the following: 1) defined objectives or research questions; 2) description of the sampling strategy, including rationale for the recruitment method, participant inclusion/exclusion criteria and the number of participants recruited; 3) detailed reporting of the data collection procedures; 4) data analysis procedures described in sufficient detail to enable replication; 5) a discussion of potential sources of bias; and 6) a discussion of limitations.

Done.

**Additional requirements, emailed subsequently.**

1) Please ensure that the funders and grant numbers match between the Financial Disclosure field and the Funding Information tab in your submission form. Note that the funders must be provided in the same order in both places as well.

- State the initials, alongside each funding source, of each author to receive each grant.

- State what role the funders took in the study. If the funders had no role in your study, please state: “The funders had no role in study design, data collection and analysis, decision to publish, or preparation of the manuscript.”

Done.

2) In your Data Availability Statement, please ensure you have provided all necessary access information in complete sentences. Access information should include the name of any data repositories used as well as digital object identifiers (DOIs), accession numbers or codes, or other persistent identifiers for all relevant data. If all data has been provided within your submission files, please use the statement "All relevant data is available within the manuscript and Supporting Information files." The Data Availability Statement will appear on the published version of your manuscript.

Done.

3) We ask that a manuscript source file is provided at Revision. Please upload your manuscript file as a .doc, .docx, .rtf or .tex.

Done.

4) Please provide separate figure files in .tif or .eps format. For more information about figure files please see our guidelines:

Done.
