# Supplementary material for: Pathways of emergency care for severely ill children in Nigerian and Ugandan hospitals: A process mapping study
Source: PLoS Med. 2026 May 6;23(5):e1004832. doi: 10.1371/journal.pmed.1004832 (PMC13166908; doi:10.1371/journal.pmed.1004832)
Supplement: Attachment — Submitted filename: Response to editors.docx [file pmed.1004832.s004.docx]

23^rd^ April 2026

Dear editors and reviewers,

We thank you for your thoughtful and thorough feedback to our manuscript.

Please find below a point-by-point response.

Kind regards,

Rami Subhi, on behalf of the authorship team.

------------------------------------------------------------

Requests from Editors:

Please note that not all of the following comments apply to your manuscript.

GENERAL EDITORIAL REQUESTS

"* At this stage, we ask that you include a short, non-technical Author Summary of your research to make findings accessible to a wide audience that includes both scientists and non-scientists. The Author Summary should immediately follow the Abstract in your revised manuscript. This text is subject to editorial change and should be distinct from the scientific abstract. Ideally each sub-heading should contain 2-3 single sentence, concise bullet points containing the most salient points from your study. In the final bullet point of ‘What Do These Findings Mean?’ Please include the main limitations of the study in non-technical language.

Thank you. This has been addressed.

Please see our author guidelines for more information: https://journals.plos.org/plosmedicine/s/revising-your-manuscript#loc-author-summary."

* Please confirm that your title complies with PLOS Medicine's style. Your title must be nondeclarative and not a question. It should begin with main concept if possible. "Effect of" should be used only if causality can be inferred, i.e., for an RCT. Please place the study design ("A randomized controlled trial," "A retrospective study," "A modelling study," etc.) in the subtitle (ie, after a colon).

Thank you. We have reviewed this and confirm the manuscript complies.

* Please confirm that your abstract complies with our requirements, including format (three sections: Background, Methods and Findings, and Conclusions) and providing all the information relevant to this study type https://journals.plos.org/plosmedicine/s/submission-guidelines#loc-abstract

Thank you. We have reviewed this and confirm the manuscript complies.

* Please ensure that the Introduction ends with a clear description of the study question or hypothesis.

Thank you. This has been addressed.

* Please ensure that all abbreviations are defined at first use throughout the text.

Thank you. Checked.

* Please confirm that all numbers presented in the abstract are present and identical to numbers presented in the main manuscript text.

Thank you. Checked.

GENERAL

* Please review your text for claims of novelty or primacy (e.g. 'for the first time') and remove this language. In addition, please check that any use of statistical terms (such as trend or significant) are supported by the data, and if not please remove them.

Checked.

* Please remove the 'conclusions' subheading from the discussion. Please also remove any other subheadings from the discussion. There should be no conclusions section. Discussion should only be referred to as such and no subheadings should be present.

Done.

* In the last sentence of the Abstract Methods and Findings section, please describe the main limitation(s) of the study's methodology.

We have added the following:

“Our findings are based on a purposive sample of facilities from Nigeria and Uganda, which may not be representative of other low-resource settings.”

* In the author summary, in the final bullet point of 'What Do These Findings Mean?', please include the main limitations of the study in non-technical language.

We have added the following:

“Our findings are based on a purposive sample of facilities from Nigeria and Uganda, which may not be representative of other low-resource settings.”

Also, please adhere to the three main sections:

"-Why Was This Study Done?

-What Did the Researchers Do and Find?

-What Do These Findings Mean?"

Done.

* Please include an Acknowledgments section in your manuscript.

We have added the following:

“We thank the staff and leadership of participating health facilities in Nigeria and Uganda, and the Ministries of Health in both countries, for their support of this study. We also acknowledge the support of the Clinton Health Access Initiative teams in Nigeria, Uganda, and globally. We are grateful to Trevor Duke for his feedback on the manuscript.”

* Please report your qualitative study according to the appropriate study design provided at http://www.equator-network.org/?post_type=eq_guidelines&eq_guidelines_study_design=qualitative-research&eq_guidelines_clinical_specialty=0&eq_guidelines_report_section=0&s= and provide the relevant completed checklist. In the checklist please include sufficient text excerpted from the manuscript to explain how you accomplished all applicable items.

We have followed the SRQR checklist. This is reported in the Methods, and detailed in Appendix S2.

* Please describe the conceptual framework underlying your qualitative analysis.

Thank you. In the Methods, we lay out our theoretical framework, drawing on a critical realist ontology and pragmatic epistemology, to describe patterns in facility organisation. We also explain the rationale for adopting inductive content analysis (Methods, p.8):

“We adopted Inductive Content Analysis (ICA) because our aim was to describe facility processes and organisations to practically guide intervention design (rather than to develop high level theories), and there are limited published data on the topic[16].”

* Please revise the text, as sentences are sometimes too dense or unclear (example paragraph 1 of the introduction, line 499, etc).

Thank you for this feedback. We have aimed to shorten the sentences and to simplify the language throughout the manuscript.

* Please remove all highlighted text.

Done.

FUNDING STATEMENT

* The funding statement should include: specific grant numbers, initials of authors who received each award, URLs to sponsors’ websites. Also, please state whether any sponsors or funders (other than the named authors) played any role in study design, data collection and analysis, the decision to publish, or preparation of the manuscript. If they had no role in the research, include this sentence: “The funders had no role in study design, data collection and analysis, decision to publish, or preparation of the manuscript.”

The statement now reads:

“This work was funded by a subgrant from the Bill and Melinda Gates Foundation (https://www.gatesfoundation.org/) and ELMA Philanthropies (https://www.elmaphilanthropies.org/) (INV-043011; HRG) awarded to HRG, and the Swedish Research Council (https://www.vr.se/english.html) (reference: 2023-01977), awarded to CK. The funders had no role in study design, data collection and analysis, decision to publish, or preparation of the manuscript.”

COMPETING INTERESTS STATEMENT

* All authors must declare their relevant competing interests per the PLOS policy, which can be seen here: https://journals.plos.org/plosmedicine/s/competing-interests For authors with ties to industry, please indicate whether any of the interests has a financial stake in the results of the current study.

Checked.

FIGURES and TABLES

*For figure S1, can you please substitute the handwritten maps with computer-made ones? It may be hard for the reader to comprehend the images. Please also note that there is a misalignment between figure letters (A,B,C) and the images.

Thank you. The purpose of Figure S1 is to illustrate the evolution from spatial to conceptual maps through the style and structure of the maps (rather than the textual content itself). We simplified the figure to include only two images (rather than three) and increased the size of each.

*In supplementary material please update the contents to reflect exactly what is shown, ie "Table S1. Deductive codes, following steps in the patient journey through a health facility, used to organise the data." Also, please remove table and figure titles and keep only the legends, which already include the title.

*For tables please place legends above the table, while for figures below the relevant figure.

Thank you. This has been addressed.

*Please, also update the order of the contents in supplementary material (and the corresponding references in the main text) as stated in the manuscript:

"Supporting information

Appendix S1

Table S1 – Deductive codes, following steps in the patient journey through a health facility, used to organise the data.

Table S2. Description of facilities that align with Archetype A

Table S3. Description of facilities that align with Archetype B

Table S4. Description of facilities that align with Archetype C

Table S5. Description of facilities that align with Archetype D

Table S6. Initial assessment, treatment and admission locations for neonates (<29 days)

Table S7. Management of ‘special’ populations: older children, trauma and surgical

conditions

Table S8. Quotes from focus group discussions with researchers, organised under categories that explain steps in the patient journey through a facility

Table S9. Characteristics of researchers participating in focus group discussions in Uganda and Nigeria

Figure S1. Example of the evolution of process maps over time

Text. Researcher positionality

Text. Process mapping data collection tool

Text. Focus group discussions"

Done.

Comments from Reviewers:

Reviewer #2: Thanks authors for their effort to improve the manuscript. I am satisfied with the response and revision. No further issues needing attention.

Thank you.

Reviewer #4: I am satisfied with the manner by which the authors have addressed my concerns. The revised manuscript is quite satisfactory.

Thank you.

Any attachments provided with reviews can be seen via the following link:

__________________________________________________

In compliance with data protection regulations, you may request that we remove your personal registration details at any time. (Use the following URL: https://www.editorialmanager.com/pmedicine/login.asp?a=r). Please contact the publication office if you have any questions.
